# A Single‐Component Adhesive Sponge Based on Blood‐Triggered and Autopenetrative Adhesion for Robust Vascular Closure

**DOI:** 10.1002/advs.202510377

**Published:** 2025-08-12

**Authors:** Rong Wang, Weishi Zheng, Yuxuan Huang, Rui Zhang, Yi Zhang, Jingying Yan, Yuqing Gu, Jingyun Xi, Tun Yuan, Hua Su, Xianzhu Zhang, Xiaozhao Wang, Shaohui Xiong, Zhenfeng Cheng, Hongwei Ouyang, Yi Hong

**Affiliations:** ^1^ Department of Sports Medicine of the Second Affiliated Hospital, and Liangzhu Laboratory Zhejiang University School of Medicine Hangzhou 310058 China; ^2^ Dr. Li Dak Sum & Yip Yio Chin Center for Stem Cells and Regenerative Medicine Zhejiang University School of Medicine Hangzhou 310058 China; ^3^ National Engineering Research Center for Biomaterials Sichuan University Chengdu Sichuan 610064 China; ^4^ Sichuan Testing Center for Biomaterials and Medical Devices Co., Ltd Chengdu Sichuan 610064 China; ^5^ Department of Pulmonary and Critical Care Medicine Regional Medical Center for National Institute of Respiratory Diseases School of Medicine Sir Run Run Shaw Hospital, Zhejiang University No. 3 Qingchun Road East Hangzhou 310016 China; ^6^ Huzhou Central Hospital Affiliated Central Hospital of Huzhou University Huzhou 313000 China; ^7^ China Orthopedic Regenerative Medicine Group (CORMed) Hangzhou China

**Keywords:** body‐fluid trigger, chain penetration, strong adhesive sponge, vascular closure device

## Abstract

Achieving strong adhesion under wet and bleeding conditions remains a major challenge for medical adhesives. Existing strategies that utilize polymer chain penetration to overcome this have shown promise but typically rely on complex external stimuli, limiting clinical use. Here, MonoSeal is introduced, a body‐fluid‐triggered adhesive sponge composed of a single oxidized polysaccharide, operating via a newly proposed mechanism termed Autopenetrative Adhesion (APA). Unlike conventional approaches, APA enables spontaneous polymer chain penetration into moist tissues via concentration‐gradient‐driven diffusion, without any external activation. This self‐driven penetration dramatically increases the interfacial density and depth of reactive groups, which then form covalent bonds with tissue amines, establishing strong tissue anchoring. Meanwhile, the proteins present in body fluids serve to cross‐link the sponge matrix, forming a rapid in situ barrier that mechanically seals the wound. By tailoring the molecular weight and sponge architecture, the dissolution–cross‐linking kinetics is fine‐tuned to optimize adhesive penetration and mechanical sealing. The resulting MonoSeal achieved robust tissue adhesion and effectively sealed the 12 Fr puncture wound on the porcine femoral artery within 30 s. When delivered via a customized applicator, MonoSeal also demonstrates promising performance as a vascular closure device following interventional access in the porcine carotid artery.

## Graphical abstract

This study presents MonoSeal, a single‐component adhesive sponge powered by the novel Autopenetrative Adhesion (APA) mechanism. Unlike traditional adhesives, which require external stimuli, APA enables strong tissue adhesion under dynamic, high‐pressure bleeding conditions. MonoSeal's ability to autonomously seal large‐size arterial defects underscores its potential for improving emergency hemostasis and vascular closure in clinical practice.

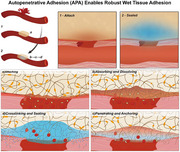

## 1 Introduction

Medical adhesives are essential adjuncts to sutures in clinical practice, commonly used to prevent postoperative bleeding, cerebrospinal fluid leakage, and gastrointestinal leakage.^[^ ^1^, ^2^, ^3^, ^4^, ^5^ ^]^ Despite their critical role, current commercial adhesives demonstrate limited adhesion to wet tissues, significantly restricting their clinical applicability.^[^ ^6^ ^]^ In recent years, wet tissue adhesives have attracted widespread attention, becoming a prominent focus of research. Among the explored strategies, chemical bonding has emerged as a particularly reliable approach.^[^ ^7^ ^]^ This method utilizes functional groups, such as succinimide or aldehyde groups, to form covalent bonds with amino or thiol groups in the extracellular matrix of tissue surfaces, enabling strong adhesion.^[^ ^8^ ^]^ However, the efficacy of adhesives depending solely on chemical bonding is fundamentally constrained by the limited availability of reactive groups on tissue surfaces, which sets an upper limit on adhesion strength.^[^ ^9^ ^]^ Additionally, wound exudates—comprising water, proteins, and cells—impede direct contact between the adhesive material and tissue surfaces or preferentially bind to the adhesive groups, thereby diminishing the likelihood of effective bonding and compromising overall adhesion performance.^[^ ^7^ ^]^ This limitation is exacerbated in cases of severe hemorrhage, where the high concentrations of proteins and cells rapidly react with the adhesive groups, effectively preventing tissue bonding.^[^ ^10^, ^11^, ^12^ ^]^

To address these limitations, physical mechanisms such as entanglement, interlocking, and permeation have been explored to complement chemical bonding through the synergistic effects of mutual impermeability and chemical covalency between polymer backbones.^[^ ^13^, ^14^, ^15^ ^]^ Central to these mechanisms is the concept of polymer penetration, wherein polymer chains not only adhere to the tissue surface but also infiltrate deeply into its microarchitecture, such as the extracellular matrix (ECM), collagen fibers, and microscopic pores or fissures present on the wound surface.^[^ ^16^, ^17^ ^]^ This deep penetration significantly increases the cross‐linking density between the adhesive polymers and the tissue matrix, while simultaneously establishing physical anchoring through interlocking and mechanical interdigitation.^[^ ^18^, ^19^ ^]^ Collectively, these interactions markedly enhance the adhesive strength and long‐term stability of the interface.^[^ ^14^, ^19^, ^20^ ^]^ However, effective polymer penetration into biological tissues often necessitates specialized treatments to facilitate polymer chain transport across tissue barriers. Techniques such as ultrasound application or mechanical force are commonly employed to promote deeper penetration of polymer chains by increasing tissue permeability and driving polymer diffusion.^[^ ^21^, ^22^ ^]^ These additional requirements impose practical limitations on the integration of this mechanism into adhesive systems, such as interventional surgery.

In addition to external stimuli, polymer dissolution serves as an intrinsic trigger for diffusion, inspiring novel strategies to incorporate penetration into traditional adhesive systems based on chemical bonding. Medical adhesive sponges are widely utilized in clinical practice as effective hemostatic materials.^[^ ^23^ ^]^ Typically, these sponges are chemically cross‐linked, resulting in a stable structure throughout usage until eventual degradation.^[^ ^24^, ^25^, ^26^, ^27^ ^]^ By lyophilizing un‐cross‐linked polymer solutions, sponge materials can be engineered to dissolve upon exposure to body fluids, releasing polymer chains that diffuse into the tissue driven by concentration gradients. Once diffused, these dissolved polymer chains may react with functional groups, such as amino or thiol groups, on the ECM, proteins, or cells within the body fluid, thereby increasing the cross‐linking density. This enhances the adhesion between the material and the tissues, allowing effective wound sealing. Furthermore, the diffusion of polymer chains into tissue pores, fibrous networks, and other microstructures facilitates physical entrapment and mechanical interlocking. These physical interactions, together with the entanglement of polymer chains within the bulk hydrogel formed on the tissue surface, contribute synergistically to enhancing adhesive strength and long‐term interfacial stability.

**Figure 1:**
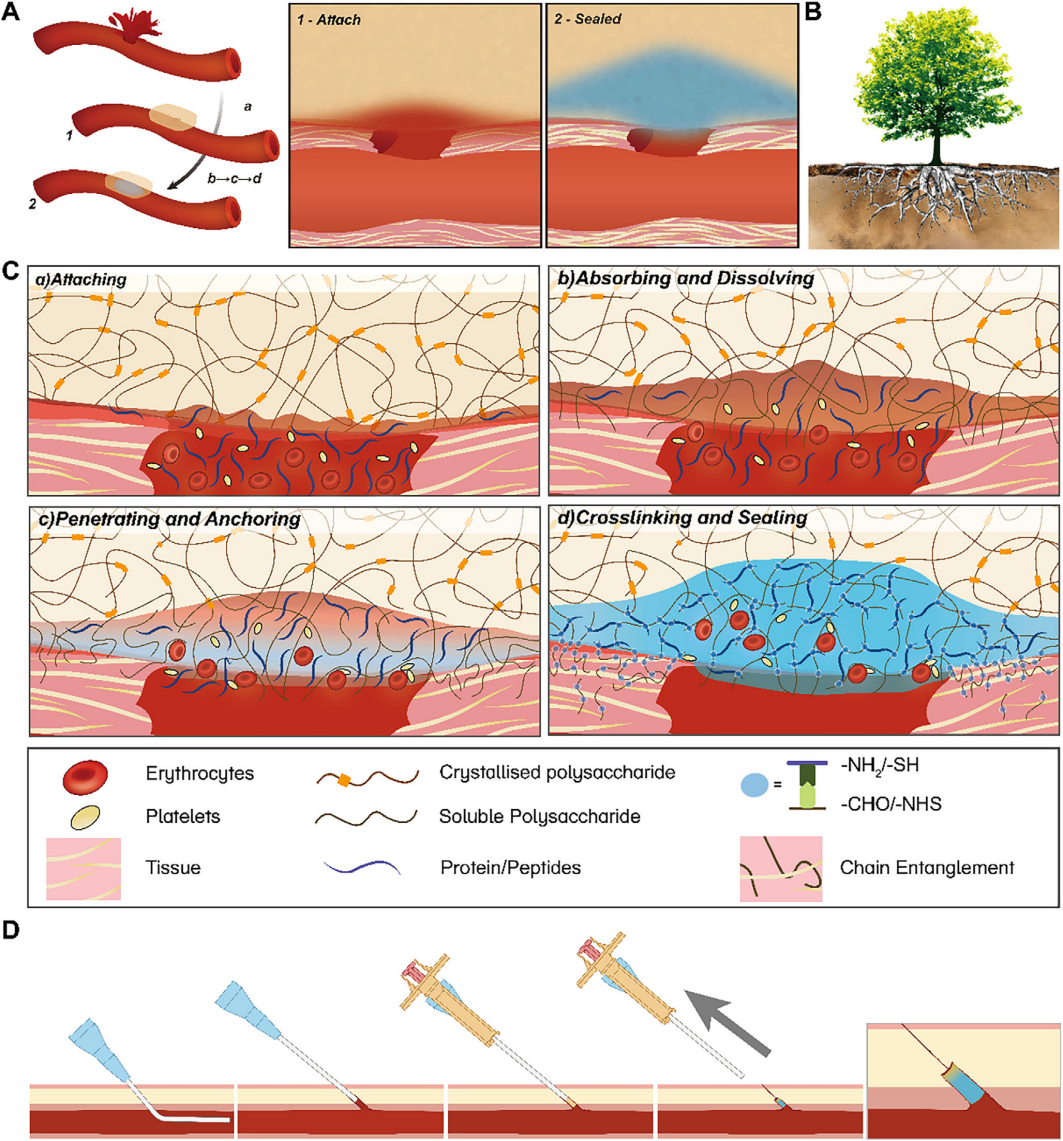
Autopenetrative Adhesion (APA) enables robust wet tissue adhesion and the development of vascular closure devices. A) Schematic illustration showing that amine‐rich body fluids, such as blood, trigger the MonoSeal sponge to form strong adhesion on the tissue surface, effectively sealing the wound. B) The analogy of tree roots penetrating deep into the ground is used to represent how the extended polymer chains of MonoSeal autonomously penetrate tissue. Similar to roots, these chains form a tight network of entanglements, improving adhesion strength through self‐penetration and covalent anchoring. C) A microscopic view illustrating the detailed mechanism of MonoSeal's adhesion. (a) Upon contact with tissue, (b) the dry sponge absorbs the body fluid and dissolves, (c) the extended aldehyde‐rich polymer chains then penetrate the tissue matrix, where they form covalent cross‐links with amine groups on the tissue surface, significantly increasing the adhesion strength, (d) establishing a firm bond that effectively seals the wound. D) Application protocols for vascular closure devices based on MonoSeal, demonstrating its potential as an effective solution for sealing vascular punctures and achieving hemostasis following interventional procedures.

Building on this rationale, we proposed a body‐fluid‐triggered, chain penetration‐enhanced adhesive sponge (MonoSeal), which is powered by the introduced Autopenetrative Adhesion (APA) mechanism (**Figure** **1**). This novel mechanism leverages the single‐component system of dissolving and penetrating interfacial tissue to achieve strong wet tissue adhesion easily (Figure 1A–C). Specifically, the sponge is composed solely of an oxidized polysaccharide, with oxidized dextran (ODex) exemplifying this approach. Our findings revealed that fine‐tuning the molecular weight of ODex is crucial for optimizing its penetration into tissue, while both molecular weight and the porous structure of the sponge collectively enhance its sealing performance. Through optimization of manufacturing parameters, MonoSeal demonstrated robust wet tissue adhesion and effectively sealed arterial injuries in porcine models, even under conditions of massive bleeding. To further evaluate its clinical potential, we designed a customized vascular closure device incorporating MonoSeal, successfully achieving hemostasis in carotid artery models following interventional procedures. Our study established MonoSeal as a safe and effective tissue adhesive material, indicating its potential for promising clinical applications (Figure 1D).

## 2 Results

### 2.1 Chain Penetration Leads to Strong Adhesion

MonoSeal is composed of a single polysaccharide. Water‐soluble polysaccharides, such as dextran (Dex), hyaluronic acid, and carboxymethylcellulose, are widely used in the production of clinical adhesives.^[^ ^28^, ^29^, ^30^ ^]^ Oxidative modification of these polysaccharides to generate aldehyde groups offers a convenient method for introducing tissue‐specific adhesive functionalities.^[^ ^31^, ^32^ ^]^ Previous studies have utilized oxidized polysaccharides to fabricate sponge‐like hemostatic agents that accelerate coagulation by enriching coagulation factors through a porous structure in the matrix.^[^ ^33^, ^34^, ^35^, ^36^ ^]^ In contrast, our study introduced MonoSeal, a body fluid‐triggered adhesive sponge enhanced by chain penetration. Unlike conventional materials that rely on passive absorption, MonoSeal actively seals wounds by dissolving upon contact with blood. The aldehyde‐rich polymer chains in the dry sponge then penetrate deep into the tissue, increasing the spatial density of reactive sites dramatically. This results in the formation of a dense, covalently cross‐linked interfacial network that extends beyond the tissue surface into the subsurface matrix, enabling rapid and robust sealing of bleeding wounds. This adhesion process is driven by a newly defined mechanism, APA, in which dissolved polymer chains, activated solely by physiological fluids, spontaneously penetrate tissue and establish extensive covalent anchoring without external stimuli. To our knowledge, this is the first study to propose and implement the APA mechanism, enabling a single‐component oxidized polysaccharide sponge to achieve strong adhesion under blood‐wet conditions and function as a standalone sealing system.

In the study, we first characterized the oxidized polysaccharide backbone. Distinct vibration peaks at 2880–2650 and 1740–1715 cm^−1^ were observed in the attenuated total reflection‐fourier transform infrared (ATR‐FTIR) spectrum of the oxidized backbone, whereas these peaks were absent in the unoxidized backbone, confirming the presence of aldehyde groups (Figure S1A, Supporting Information). In addition, by adjusting the ratios of Dex and potassium permanganate, we observed that the resulting ODex displayed a distinct absorption peak at 238 nm in the ultraviolet (UV) spectrum, with the peak intensity being highest at a 1:1 reaction ratio (Figure S1B, Supporting Information). This indicates that the maximum number of aldehyde groups was incorporated into ODex. To ensure a sufficient number of aldehyde groups were available for strong adhesion, we selected the sample with the highest aldehyde content for subsequent investigations. The degree of aldehyde substitution was calculated as 56.8% through hydroxylamine hydrochloride titration.

**Figure 2:**
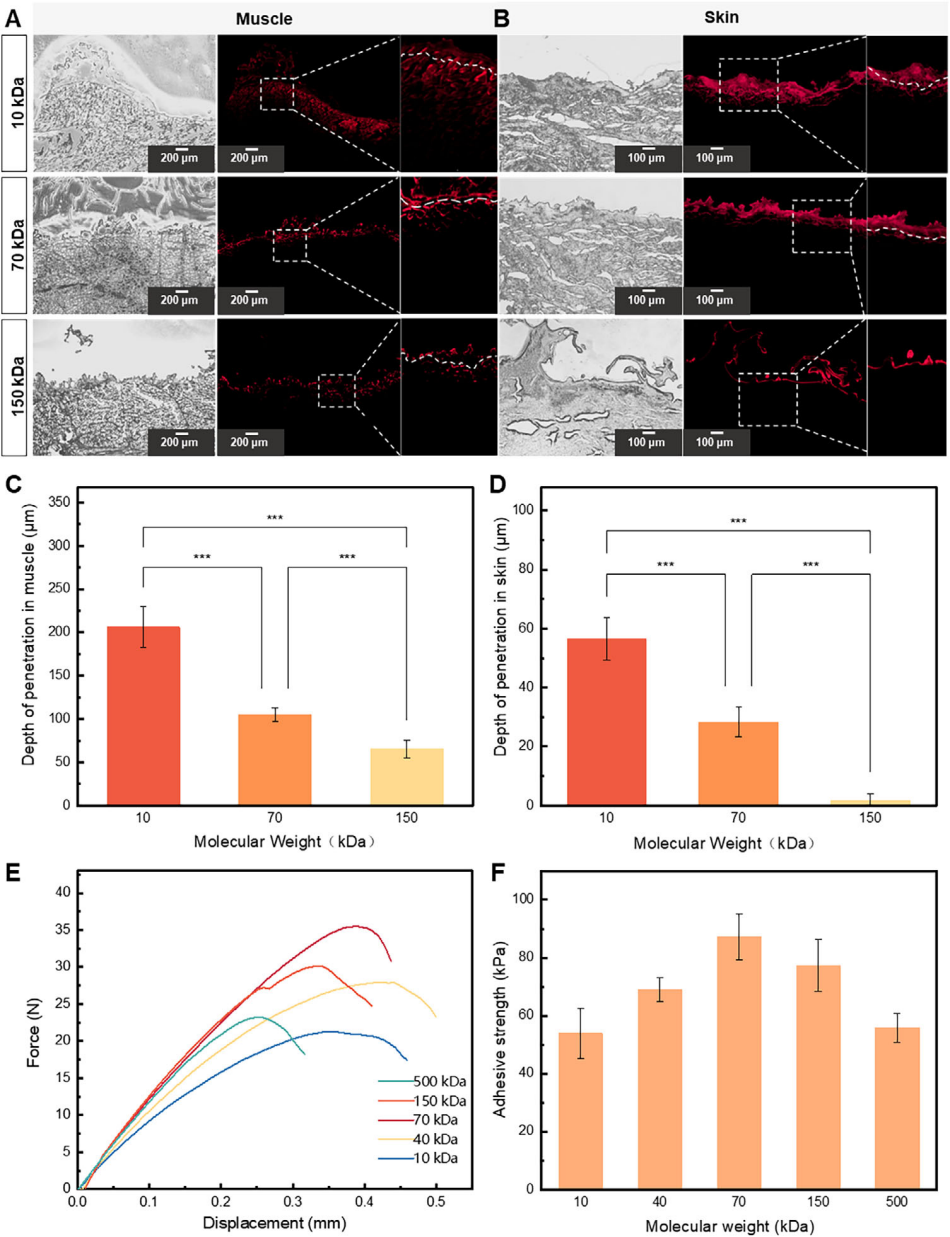
Chain penetration and subsequent interfacial adhesion are modulated by polymer molecular weight. A,B) Optical and fluorescent images showing the penetration of oxidized dextran in MonoSeal with different pore structures (red stain: RB‐PEG‐NH2 modified oxidized dextran). C,D) Quantitative analysis of the penetration depth of MonoSeal with varying molecular weights in different tissues, showing that larger ODex molecules penetrate less deeply. E) Force‐displacement curves obtained from lap shear tests of MonoSeal with varying ODex molecular weights. F) Quantitative analysis of the adhesion strength of MonoSeal with varying ODex molecular weights, showing that adhesion strength peaks at 70 kDa molecular weight. (n = 3, Data are presented as mean ± SD, * *p* < 0.05, ** *p* < 0.01, *** *p* < 0.001).

We subsequently investigated whether ODex is capable of penetrating tissues through dissolution and diffusion. Various molecular weight forms of ODex were fluorescently labeled, and their penetration in skin and muscle tissues was assessed (**Figure** **2**A,B). We observed that the penetration depth of ODex was inversely related to its molecular weight, with larger molecules penetrating less deeply (Figure 2C,D). Furthermore, ODex penetrated muscle tissue significantly more deeply than skin tissue at the same molecular weight, likely due to the denser nature of the skin. These findings indicate that ODex can penetrate tissues deeply via dissolution‐diffusion, providing the potential for forming chain penetration‐enhanced adhesion.

For this reason, we then explored the effect of ODex penetration depth on the MonoSeal adhesion properties through mechanical testing. The results showed that as the molecular weight of ODex increased from 10 to 500 kDa, the adhesion strength of MonoSeal initially increased and then decreased (Figure 2E,F). The greatest adhesion strength was observed at a molecular weight of 70 kDa, reaching 87.21 kPa. This phenomenon can likely be explained by the trade‐off of bulk mechanics of MonoSeal, determined mainly by chemical cross‐linking and its physical interaction with tissue. On one hand, despite the fact that smaller molecular weight ODex penetrated deeper into the tissue, it cannot form a dense polymer network following cross‐linking with proteins in body fluid, resulting in poor bulk mechanics. On the other hand, larger molecular weight ODex, although forming a denser network with the MonoSeal matrix, penetrates less deeply, thus not providing sufficient cross‐linking sites in deeper tissues, resulting in sub‐optimal interfacial connection strength. Based on these results, a molecular weight of 70 kDa appears to be the optimal point where penetration depth and the strength of the polymer network with the MonoSeal matrix are balanced. The penetration of the polysaccharide chains significantly strengthens the interfacial integration between MonoSeal and wet tissues. During the process, dissolved polysaccharide chains penetrate the tissue interface, increasing the covalent cross‐linking density, and conjugate with the hydrogel matrix formed at the interface, thereby enhancing the adhesion performance of the interface. This mechanism allows the molecular chains of MonoSeal to anchor deeply within the tissue, much like tree roots penetrating soil to establish a firm grip (Figure 1B). Notably, unlike conventional chain penetration strategies require external energy inputs such as ultrasound or heat to enhance interfacial bonding,^[^ ^14^, ^21^, ^37^ ^]^ our approach enables strong adhesion solely through the dissolution and chain penetration within a short time under physiological conditions. This autonomous process is the hallmark of the APA mechanism, where body fluids alone trigger rapid tissue adhesion.

### 2.2 Adhesive Performance and Closure Effect Verified In Vitro

**Figure 3:**
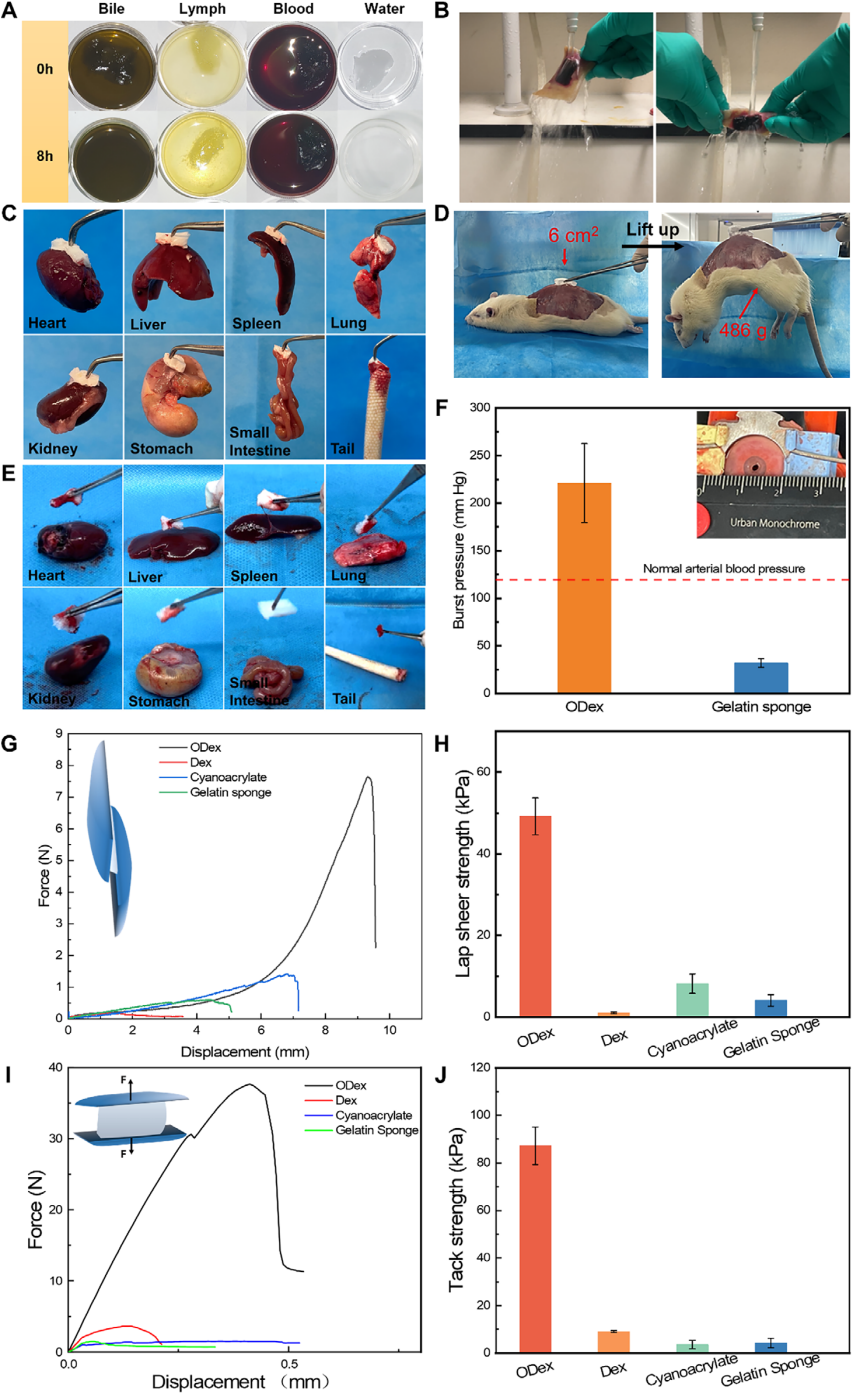
Body fluid triggered gelation and adhesion of MonoSeal in vitro. A) Gel formation of MonoSeal in different body fluids and water. MonoSeal rapidly forms a hydrogel in protein‐rich liquids (blood and lymph) but dissolves in protein‐poor liquids (bile and water). B) MonoSeal exhibits robust adhesion to tissue, capable of withstanding external forces such as stretching, twisting, and water impact. C) MonoSeal forms strong adhesion to various soft tissues, allowing them to be lifted with ease. D) MonoSeal firmly adheres to muscle surfaces, lifting a 486 g rat. E) Gelatin sponge fails to adhere to body fluid‐covered tissue. F) Burst pressure comparison between MonoSeal and gelatin sponge. G,H) Lap shear test results for MonoSeal and commercial adhesives. I,J) Tack test results for MonoSeal and commercial adhesives.

The strategy we have devised offers distinct advantages, not only in enabling ODex to establish robust adhesion in body fluids via a dissolution‐diffusion‐cross‐linking mechanism but also in facilitating the removal of unreacted ODex sponge using fluids that do not induce cross‐linking of ODex. During this process, the reaction between the aldehyde groups on ODex and the amino groups of proteins present in body fluids contributes to the gelation and adhesion properties of MonoSeal. To assess the conditions under which MonoSeal can gel, the material was immersed in fresh bile, lymphatic fluid, blood, and water at 37 °C, and its behavior was subsequently observed (**Figure** **3**A).

Lymphatic fluid and blood, rich in proteins such as plasma proteins and hemoglobin, promoted rapid gel formation. MonoSeal quickly absorbed these fluids, forming stable hydrogels. In contrast, MonoSeal dissolved over time when exposed to water, due to the lack of amino groups from organic molecules. Notably, MonoSeal does not gel in fresh bile, which aligns with our hypothesis that the presence of amino groups on proteins in bodily fluids is essential for the gelation of MonoSeal. Proteins constitute only 4.5% of bile, a concentration significantly lower than that of plasma proteins in blood (54%).^[^ ^38^, ^39^ ^]^ Consequently, bile lacks sufficient protein content to act as an effective cross‐linking agent for MonoSeal gelation. These findings indicated the potential of MonoSeal for clinical wound closure. After forming a strong adhesion with amino‐rich body fluids, the non‐reacted sponge material can be easily removed with saline, offering both strong adhesion and simple removal.

Subsequently, MonoSeal was applied to various tissue surfaces, and its adhesive effect was evaluated by lifting the tissue with tweezers and comparing this to that of medical gelatine sponges. While medical gelatin sponges demonstrated an inferior adhesion property, MonoSeal showed strong adhesion by binding to the tissue surface via blood at the tissue interface (Figure 3C,E). The heart, liver, spleen, and lungs of rats could be easily lifted after adhesion formation. Notably, MonoSeal also formed strong bonds on muscle surfaces, lifting a 486 g rat (Figure 3D; and Movie S1, Supporting Information). This strong bonding is likely attributed to the superficial layer of MonoSeal dissolving upon contact with water on the tissue surface, enabling the dissolved ODex to penetrate the tissue, tangling with microporous structures, providing abundant cross‐linking sites to establish covalent cross‐linking networks with amino components like proteins in the tissue, thereby enhancing adhesion. Additionally, the robust adhesive layer remained visible on the muscle surface, even after MonoSeal was removed with tweezers (Figure S2B, Supporting Information). These results demonstrated MonoSeal's potential for hemostasis and anti‐leakage in various tissues.

Besides, the interface formed between MonoSeal and tissues is exceptionally robust, withstanding external forces including stretching, twisting, and impact (Figure 3B). These characteristics underscore its substantial potential for use in internal organ closure and the prevention of leakage. To systematically assess adhesive performance, we conducted lap shear tests and tack adhesion tests under wet tissue conditions and compared MonoSeal to representative commercial adhesives (Figure 3G–J). MonoSeal demonstrated significantly superior lap shear strength (49.18 kPa) compared to cyanoacrylate (8.16 kPa) and gelatin sponge (4.07 kPa), reflecting its robust interfacial bonding behavior. The superiority of MonoSeal was further validated by tack strength tests on blood‐wetted tissue surfaces, in which MonoSeal, cyanoacrylate adhesive, and gelatin sponge displayed tack strengths of 87.21, 3.59, and 4.31 kPa, respectively. The interfacial failure observed in the assay (Figure S2E, Supporting Information) provided further mechanistic insights: the Dex sponge dissolved rapidly upon contact with blood, resulting in minimal adhesion dominated by weak noncovalent interactions; gelatin sponges and cyanoacrylate adhesives failed by interfacial detachment at tissue‐material boundaries, suggesting a poor interfacial anchorage. These failures can be attributed to the inability of conventional adhesives to establish robust chemical bonding under physiological conditions. Specifically, although cyanoacrylate adhesives have strong adhesion in a dry environment, their adhesion is significantly reduced in the presence of water and blood because the cyanoacrylate monomers cure rapidly upon contact with a water layer and are unable to form an effective bond with the tissue surface. For gelatin‐based materials, certain hydrogen bonds can be formed on the relatively dry tissue wound surface. However, in the presence of protein‐rich fluids like blood, these interactions are significantly weakened or competitively disrupted. The abundance of serum proteins and the formation of a hydrated interfacial layer prevent direct contact between the gelatin matrix and tissue‐bound functional groups, thereby limiting adhesive strength. By contrast, MonoSeal exhibited a distinct failure mode characterized by cohesive rupture within the sponge matrix, rather than detachment at the material–tissue interface. Such a failure mode further confirms that MonoSeal, which forms a highly reinforced interface due to chain penetration‐enhanced dense covalent cross‐linking, is firmly anchored to tissue in the blood.

To assess the mechanical properties and closure effectiveness of MonoSeal in practical scenarios, we evaluated its bursting pressure by adhering each material to an enteric coating with a 2 mm diameter hole exposed to fresh blood. The results showed that MonoSeal achieved a burst pressure of 221.11 mmHg, significantly exceeding that of gelatin sponges (32 mmHg) and surpassing typical human systolic blood pressure (60–160 mmHg) (Figure 3F; Figure S2C,D and Movies S2 and S3, Supporting Information), indicating its capability to effectively seal bleeding sites. These results indicated that the interfacial adhesion was significantly strengthened by the dense network of the penetration of polysaccharide chains, enabling MonoSeal with sufficient adhesive strength and mechanical integrity to withstand human blood pressure, underscoring its potential for sealing arterial and other severe hemorrhages.

### 2.3 Superior Hemocompatibility and Biocompatibility

MonoSeal rapidly converts from a porous sponge to a hydrogel upon contact with amino‐rich body fluids, increasing its retention time in the body. It is vital to ensure that MonoSeal, its hydrogels, and degradation products do not cause acute tissue damage, inflammation, or other adverse reactions during in vivo closure. The hemocompatibility of MonoSeal was assessed by incubation with erythrocytes (Figure S3A, Supporting Information). MonoSeal‐treated supernatant was clear and transparent after incubated with erythrocytes for 1 h, and its hemolysis rate was only 0.14%. This indicated that MonoSeal did not cause obvious rupture or hemolysis of erythrocytes during contact with blood, showing excellent blood compatibility. Meanwhile, MonoSeal was pre‐formed into hydrogel, and L929 cells were cultured for 24 h with gel extracts and standard medium to assess cell viability and proliferation (Figure S3B, Supporting Information). The results showed that the extract of MonoSeal didn't exert a significant impact on cell activity, with L929 cell survival rates exceeding 95%, suggesting great biocompatibility of MonoSeal.

To assess the biocompatibility of MonoSeal in vivo, we implanted MonoSeal under the skin of rats and monitored its degradation, followed by histological analysis (Figure S3C,D, Supporting Information). As MonoSeal was not cross‐linked before implantation, it rapidly dissolved in subcutaneous body fluid mainly containing water and electrolytes, owing to the low amino content in rat percutaneous body fluids. Unlike commercial gelatin sponges, MonoSeal exhibited nearly complete dissolution, with no residual gel observed three days after implantation. Despite the rapid degradation, histological results showed only mild inflammatory cell infiltration, significantly lower than that induced by gelatin sponges. These findings indicate that the degradation of non‐cross‐linked MonoSeal proceeds in a manner that is both safe and well‐tolerated in vivo.

Subsequently, to assess the in vivo performance of cross‐linked MonoSeal, we evaluated its biocompatibility and degradation in a rabbit liver hemorrhage model, where cross‐linking occurred in situ upon contact with blood (Figure S3E, Supporting Information). Upon full blood contact, MonoSeal rapidly cross‐linked and formed a physical barrier at the wound site, preventing further bleeding. Sponges with insufficient blood exposure are gradually degraded by surrounding body fluids over time. Histological analysis revealed a reduction in inflammatory cells at the wound, with almost complete resolution by day 7. These results strongly demonstrate that cross‐linked MonoSeal maintains excellent biocompatibility throughout in vivo degradation. Notably, the physical barrier formed by MonoSeal withstood liver tissue contraction and stretching during degradation, indicating a tissue‐matched mechanical property of the formed hydrogel.

Collectively, these data indicated that MonoSeal exhibits good biocompatibility in both non‐cross‐linked and blood‐cross‐linked states, with distinct but favorable degradation behaviors suitable for various in vivo applications.

### 2.4 Effect of Pore Structure on Sealing Performance

**Figure 4:**
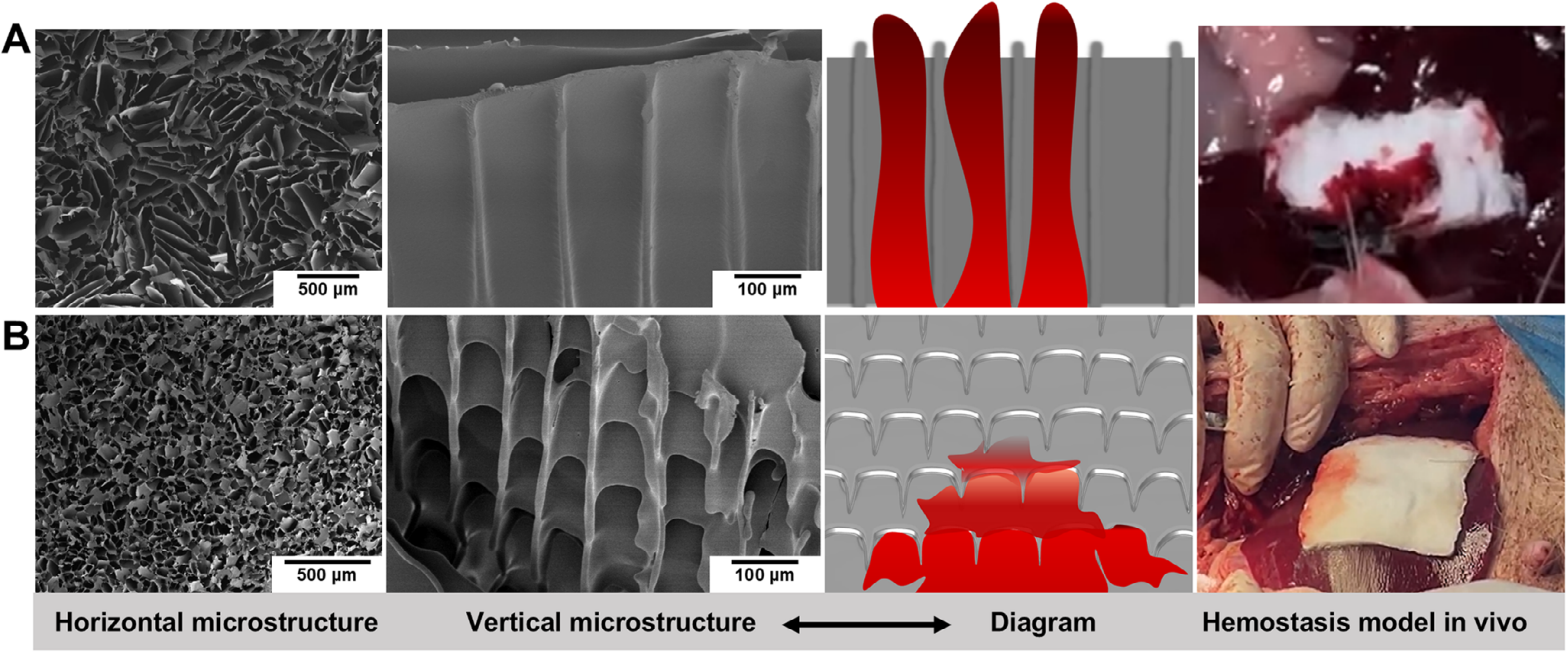
Microstructure significantly influences the sealing performance of MonoSeal. A) SEM images, schematic diagrams, and digital photos showing MonoSeal with a directional pore structure, which exhibited poor sealing performance and failed to seal liver hemorrhage in rabbits. B) SEM images, schematic diagrams, and digital photos show MonoSeal with a non‐directional pore structure, providing superior sealing performance and enabling rapid sealing of porcine liver hemorrhage.

In vitro experiments demonstrated that MonoSeal exhibits exceptional adhesive and sealing properties. However, in vivo testing under severe hemorrhagic conditions revealed that these interfacial properties alone were insufficient to ensure effective hemostasis. While MonoSeal firmly adhered to tissue surfaces, it failed to prevent blood breakthrough under high‐pressure bleeding, resulting in sealing failure (**Figure** **4**A). This failure indicated that effective hemostasis requires not only superior interfacial adhesion but also an optimized internal architecture to resist fluid infiltration. We proposed that the failure of MonoSeal under high‐pressure bleeding is attributed to the directional pore structure formed during freeze‐drying (Figure 4A). The sublimation of ice crystals produced anisotropic pore channels along the thickness direction, which aligned with the blood flow path. This alignment enabled pressurized blood to infiltrate faster than ODex could dissolve and cross‐link, disrupting gelation and causing premature failure.

To address this issue, we systematically optimized the sponge's pore architecture by adjusting freeze‐drying parameters, specifically freezing temperature and polymer concentration, to reduce ice crystal size and eliminate directional alignment (Figures S4 and S5, Supporting Information).

At a freezing temperature of ‐80 °C and a polymer concentration of 7–8%, MonoSeal exhibited a highly compact and isotropic porous structure, with significantly reduced pore size and suppressed directional alignment. The smaller ice crystals formed at lower temperature and higher solution concentration minimized anisotropic sublimation tracks, while simultaneously promoting the emergence of transverse, wave‐like barrier features throughout the sponge. These microstructural features acted as mechanical “check valves” that prevent upward blood infiltration, ensuring that the rate of ODex dissolution and gelation exceeded the rate of blood infiltration. In contrast, lower ODex concentrations (e.g., 5%) produced wider, vertically aligned pores that lacked structural barriers and failed to delay fluid infiltration (Figure S4B,C, Supporting Information). Comparative scanning electron microscopy (SEM) analysis further confirmed that sponges fabricated under ‐80 °C/8% conditions exhibited narrow pore size distribution (≈77.07 µm), increased homogeneity, and reduced anisotropy, which was conducive to enhanced mechanical sealing and fluid‐triggered gel formation.

Building on these structural optimizations, we proceeded to evaluate the in vivo sealing performance of the enhanced structure. The optimized MonoSeal (‐80 °C/8%) achieved rapid and effective hemostasis in both rabbit and porcine liver hemorrhage models, with no subsequent sealing failures observed (Figure 4B; Figure S4F,G and Movies S4 and S5, Supporting Information). In contrast, the radial‐oriented MonoSeal (‐80 °C/5%) experienced rupture and continuous bleeding in rabbit liver hemostasis (Figure S4F and Movie S4, Supporting Information). This performance difference can be attributed to the dense network that limits the depth of blood infiltration, while the transverse undulations delay fluid propagation, allowing ODex sufficient time for localized dissolution, penetration, and covalent gelation with the tissue.

Collectively, these results emphasize the critical role of microstructure in determining bulk sealing behavior. The integration of dense, isotropic pore networks with directional barrier geometry directly improves the coordination between fluid absorption, chain penetration, and interfacial bonding, establishing an essential structure‐function paradigm for MonoSeal under dynamic bleeding conditions.

### 2.5 Post‐Interventional Hemostasis In Vivo

The demand for effective post‐interventional vascular closure has grown alongside the widespread adoption of endovascular procedures.^[^ ^40^, ^41^, ^42^ ^]^ Transarterial puncture remains the most common access route, and achieving rapid and secure hemostasis at the puncture site is a critical step to reduce complications and enhance patient outcomes. Manual compression of puncture points currently remains widely used in clinics for vascular closure.^[^ ^43^ ^]^ Typically, continuous pressure is applied to the puncture site for 10–20 min, followed by local pressure fixation, with bed rest lasting 4–6 h.^[^ ^44^ ^]^ However, this method has notable drawbacks, including imprecise pressure application, prolonged compression, patient discomfort, extended bed rest, and increased risk of postoperative complications for both patients and healthcare professionals.^[^ ^43^, ^45^ ^]^ Moreover, the increasing demand for large‐diameter vascular sheaths in complex interventions further complicates puncture‐site hemostasis due to the larger wound area and higher blood flow rates. To address the extended time required for manual compression, vascular closure devices like Angio‐Seal and Perclose vascular staplers have been developed to close puncture sites using biomaterials or sutures, shortening hemostatic and bed rest times to 4–10 min and 1–4 h, respectively.^[^ ^46^ ^]^ However, these devices still exhibit significant clinical limitations, such as procedural complexity, material retention, and risks of stenosis and pseudoaneurysms.^[^ ^47^, ^48^ ^]^ In light of the superior sealing efficiency and biocompatibility of our MonoSeal, we demonstrated its use as a novel vascular closure device for the efficient and rapid post‐interventional closure of puncture sites, capable of addressing both large and small puncture wounds.

**Figure 5:**
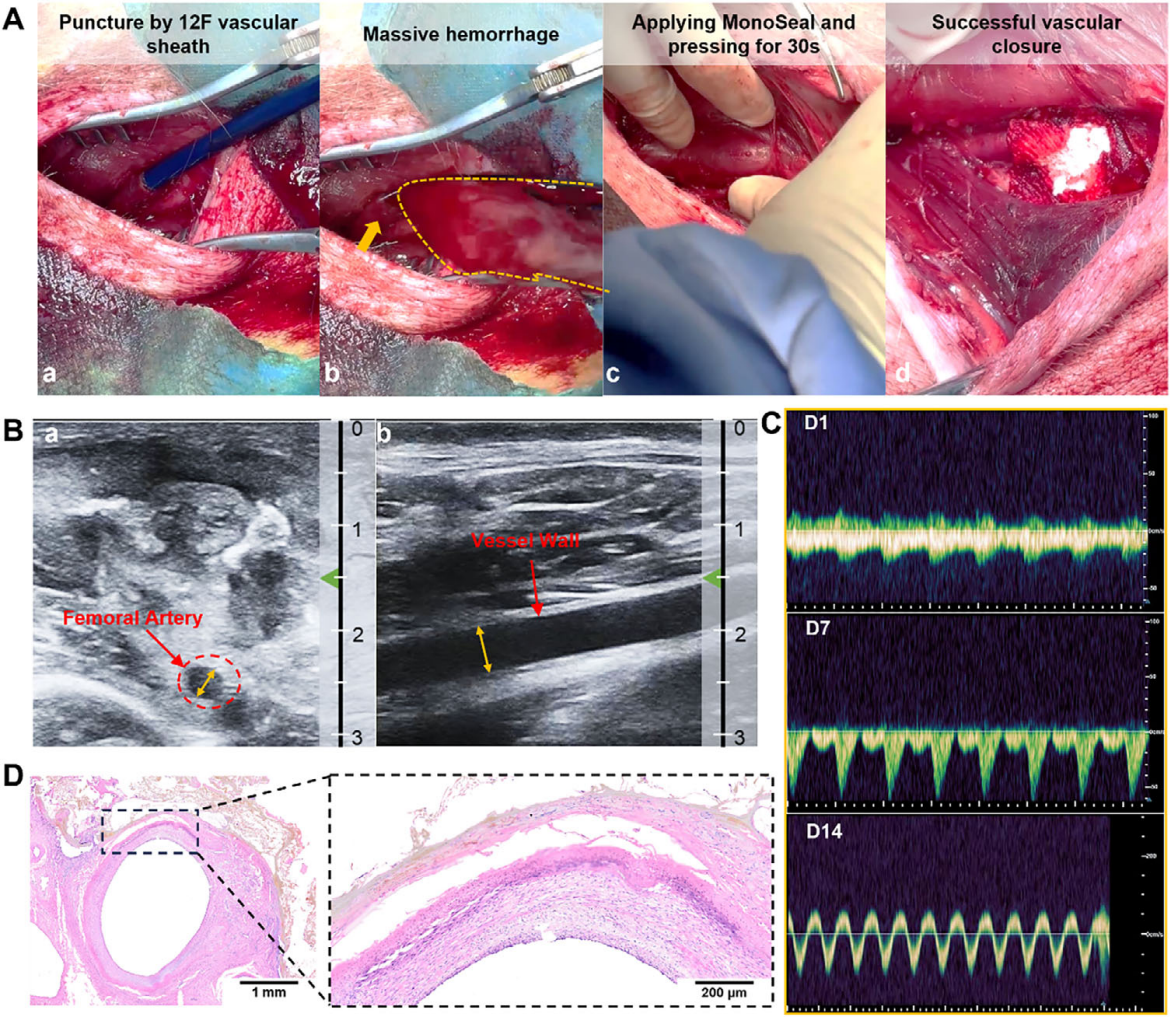
MonoSeal rapidly arrests lethal hemorrhage on a porcine femoral artery during an open surgery. A) Digital photos showing the application process of MonoSeal on a porcine femoral artery following the puncture of a large‐sized vascular sheath. B) Ultrasound images demonstrating femoral artery patency 1 day post‐procedure (Depth unit: cm). C) Blood flow volume through the porcine femoral artery at 1, 7, and 14 days after surgery. D) H&E staining analysis of the femoral artery at 2 weeks post‐procedure, showing tissue response and healing.

To verify the sealing efficacy of MonoSeal in a large‐size puncture site, we simulated a lethal interventional trauma in the porcine femoral artery using a 12 Fr vascular sheath set. Upon sheath withdrawal, a sizable puncture defect formed on the vascular surface. Blood then gushed out from the puncture point, creating a strong blood flow. Despite proximal vascular compression, uncontrolled bleeding persisted, rapidly obscuring the surgical field. MonoSeal was immediately applied to the bleeding site and compressed persistently for 30 s, achieving complete hemostasis successfully. Residual surface blood was subsequently cleared with sterile gauze, and the hemostatic site was monitored for 5 min, with no further bleeding or hematoma formation (**Figure** **5**A; and Movie S6, Supporting Information).

After applying MonoSeal for hemostasis, the blood flow and vascular recovery of the femoral artery were assessed. Doppler ultrasound imaging of the femoral artery was performed on day 1 postoperatively. The results showed a clear femoral artery wall structure, and the vessel remained patent (Figure 5B; and Movie S8, Supporting Information). MonoSeal effectively sealed the femoral artery trauma without any blood leakage. Blood flow gradually normalized, and hemodynamic status improved with prolonged treatment (Figure 5C), indicating that the vessel healed without stenosis or thrombosis. At 2 weeks post‐surgery, histological analysis of the puncture site revealed that the healed vessels exhibited a normal three‐layered structure, with well‐preserved endothelial, medial, and adventitial layers, with defect position distinguishable from hematoxylin and eosin (H&E) image (Figure 5D). Abnormal thrombus, pseudoaneurysm, or arterial entrapment was not observed in the vessel wall. The MonoSeal adhesive integrated well with the vascular tissue, with minimal necrosis or inflammation at the wound site, further confirming its biosafety and effective occlusive repair.

**Figure 6:**
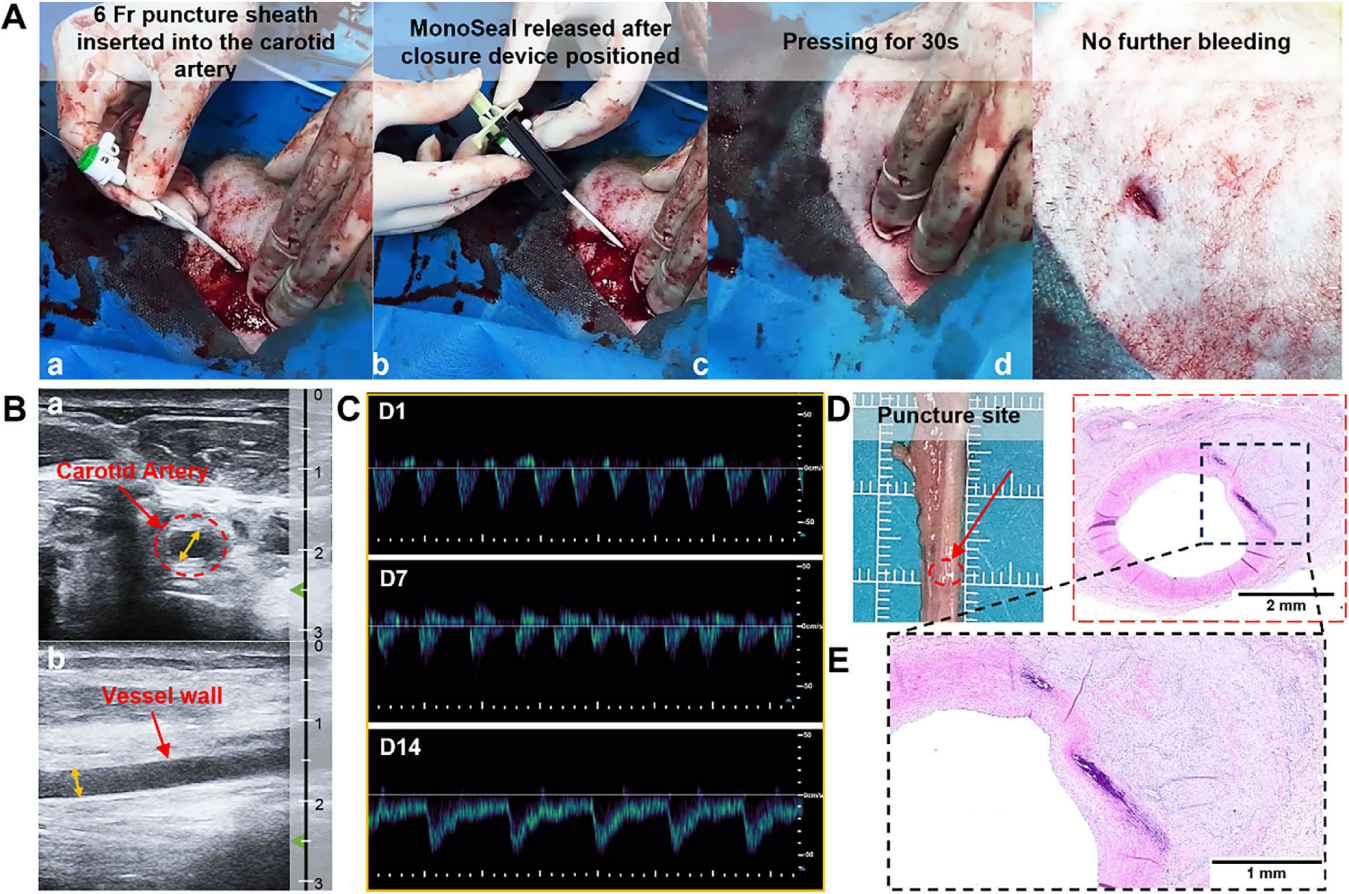
MonoSeal‐based vascular closure device rapidly achieves hemostasis on the porcine carotid artery following intervention. A) Digital photos showing the application procedure of the MonoSeal‐based vascular closure device during porcine carotid artery intervention surgery. B) Ultrasound images demonstrating carotid artery patency 1 day post‐procedure (Depth unit: cm). C) Blood flow volume through the porcine carotid artery at 1, 7, and 14 days after surgery. D) Gross view of the repaired carotid artery at 2 weeks post‐procedure. E) H&E staining analysis of the carotid artery at 2 weeks post‐procedure, showing tissue response and healing at the site of closure.

To simulate clinical deployment and broaden the translational applicability of MonoSeal, we designed a customized delivery device to store MonoSeal and apply it in a minimally invasive manner (Figures S6 and S7, Supporting Information). The snap‐lock mechanism allowed secure positioning and simplified operation. Using this system, we mimicked clinical vascular interventions in porcine carotid arteries using a 6 Fr catheter. Upon sheath withdrawal, rapid and forceful bleeding occurred at the puncture site. The MonoSeal‐based vascular closure device was positioned directly through the residual sheath positioned at the puncture site. The catheter sheath was then quickly locked into the blocker, and the occluder plug was unlocked before MonoSeal was pushed to the vascular puncture site, and all devices were withdrawn. Carotid hemostasis was completed by pressing the puncture sites for 30 s. The residual blood around the puncture site was then cleared using sterile gauze, and the porcine was monitored for 5 min. There was no blood leakage at the puncture site, and the porcine physiological state was gradually stabilized, with breathing returning to normal (**Figure** **6**A; and Movie S7, Supporting Information).

Blood flow, hematoma, and vascular recovery were monitored following the application of MonoSeal. Doppler ultrasound and CT imaging of the carotid artery were performed 1 day after surgery. Results showed a clear wall structure, with the carotid arteries remaining patent and intact (Figure 6B; and Movie S9, Supporting Information). No significant blood oozing or hematoma formation was observed (Figure S8, Supporting Information). Carotid blood flow gradually normalized with extended treatment. At 2 weeks post‐surgery, hemodynamic status was fully restored, with no signs of flow disturbances or stenosis (Figure 6C). Histological analysis of the puncture site revealed that the carotid injury site had largely healed, with the vessel displaying a structure similar to a normal artery (Figure 6D,E). These findings strongly support MonoSeal as a promising therapeutic material for post‐interventional hemostasis, with the potential to serve as a next‐generation bioadhesive closure solution in both large‐ and small‐caliber vascular interventions.

## 3 Discussion

In this study, we developed MonoSeal, a chain penetration‐enhanced, body fluid‐triggered adhesive sponge with water‐soluble modified polysaccharide as the sole component. MonoSeal leverages a newly defined mechanism, APA, whereby dissolved polymer chains spontaneously penetrate tissue and establish dense covalent bonding under physiological conditions. Unlike traditional tissue adhesives that rely on external stimuli to promote adhesion, MonoSeal functions autonomously. It achieves rapid and robust wet tissue adhesion solely via self‐activated dissolution, diffusion, and covalent anchoring of polymer chains upon contact with body fluids (Figure S9, Supporting Information). This work contributes to both theoretical advancements in material science and practical convenience in manufacturing and commercialization.

Oxidized polysaccharides have been widely used as functional platforms in various hemostatic and adhesive biomaterials due to their abundant aldehyde groups. However, currently oxidized polysaccharide‐based sponges primarily function as coagulation‐promoting matrices, relying on passive fluid absorption and porous enrichment to accelerate platelet activation or fibrin formation.^[^ ^49^, ^50^ ^]^ These systems often suffer from poor control over interfacial bonding, particularly under wet and hemorrhagic conditions, where rapid dilution and insufficient diffusion hinder effective covalent anchoring. To strengthen material–tissue adhesion, strategies aimed at increasing cross‐linking density and penetration depth through polymer chain diffusion have attracted considerable attention. Nevertheless, prior studies primarily relied on non‐physiological cues to provide energy to promote polymer chain penetration into tissues, limiting their practicality in emergent clinical scenarios where rapid, equipment‐free operation is essential. For instance, the topohesion approach employs pH modulation to transition polymer chains between diffusion and cross‐linking states, facilitating their infiltration into the adhesive interface to enhance adhesion.^[^ ^51^ ^]^ Similarly, ultrasound‐induced strategies leverage external forces to drive adhesion materials deeper into tissue structures rather than confining them to the surface.^[^ ^21^ ^]^ By contrast, the APA mechanism employed by MonoSeal represents a fundamental shift. Upon contact with body fluids like blood, MonoSeal's dry sponge matrix dissolves, releasing extended aldehyde‐rich polysaccharide chains that spontaneously penetrate the tissue, akin to roots penetrating soil. This autonomous penetration dramatically increases the spatial density of reactive sites, creating a dense, covalently cross‐linked interfacial hydrogel network that firmly anchors the sponge to both the tissue surface and subsurface layers. The APA mechanism is entirely driven by physiological conditions, enabling rapid, robust tissue adhesion under dynamic bleeding conditions, without requiring external energy sources. This novel mechanism provides a powerful and efficient alternative to traditional approaches that rely on external energy inputs, representing a new paradigm for tissue sealing.

Building upon the APA mechanism, we further optimized the microstructural design of MonoSeal to synchronize polymer chain diffusion kinetics with physiological demands. Given that rapid dissolution and deep chain penetration are all prerequisites for effective APA‐driven adhesion, and that the sponge microstructure critically governs macroscopic sealing performance, both the molecular weight of oxidized dextran and the freeze‐dried sponge architecture were systematically tuned. Lower molecular weight facilitated faster dissolution and deeper chain infiltration, but compromised bulk mechanical integrity, while higher molecular weight enhanced internal network strength at the cost of penetration efficiency. A molecular weight of 70 kDa was identified as the optimal balance, enabling sufficient tissue penetration without sacrificing structural robustness. Simultaneously, the freeze‐drying process was tailored to refine vertical pore structures, which otherwise compromised sealing integrity by facilitating rapid blood infiltration before adequate polymer dissolution and interfacial anchoring could occur. By optimizing pre‐freezing temperature and polymer concentration, a compact, isotropic porous architecture with transverse wave‐like features was achieved, minimizing vertical blood infiltration and prolonging the interaction time for polymer diffusion and gelation. This microarchitecture coordinates with the APA mechanism, ensuring that chain dissolution, penetration, and covalent anchoring occur rapidly and efficiently even under high‐pressure bleeding conditions. Thus, MonoSeal's structural engineering complements its molecular‐scale adhesion mechanism, establishing a coherent design from molecular interactions to macroscopic performance for achieving robust vascular closure.

In addition to its contributions to material research, the MonoSeal developed in this study represents a paradigm shift in vascular closure technologies, addressing longstanding limitations of current clinical approaches. Commercially available vascular closure devices, such as Angio‐Seal and ExoSeal, face significant limitations, including the risk of residual material deposition and poor applicability for large vascular punctures.^[^ ^52^, ^53^, ^54^ ^]^ To address these challenges, adhesion‐based vascular closure devices, such as MynxGrip, have been investigated.^[^ ^54^, ^55^, ^56^ ^]^ However, their insufficient adhesive properties restrict their use following large arterial sheath interventions. To date, no existing solutions demonstrate the capacity to achieve reliable closure of large vascular punctures via external adhesion alone. In this context, MonoSeal, with its innovative APA mechanism and optimized microstructure, overcomes these challenges. Large animal studies demonstrated its efficacy in sealing both large open‐surgery femoral artery defects (12 Fr) and clinically relevant carotid artery punctures (6 Fr) following interventional procedures. This exceptional sealing performance, achieved without auxiliary equipment or the need for local blood coagulation, indicates the unique advantages of MonoSeal over existing closure technologies. Future work focuses on large‐size interventional closure simulations, which are critical for MonoSeal to fully realize its full clinical potential.

## 4 Conclusion

In summary, this study proposes MonoSeal, a body fluid‐triggered adhesive sponge composed of a single water‐soluble modified polysaccharide. It operates through a newly defined mechanism, APA, which leverages physiological cues rather than external stimuli, to achieve robust wet‐tissue adhesion. MonoSeal's unique approach combines spontaneous dissolution, chain penetration, and covalent anchoring, enabling rapid and durable sealing even under challenging hemorrhagic conditions. Through systematic optimization of polysaccharide molecular weight and freeze‐dried porous architecture, MonoSeal effectively synchronizes polymer chain diffusion kinetics with physiological demands. This ensures rapid activation, deep tissue infiltration, and mechanical robustness, all while maintaining batch‐to‐batch consistency and commercial viability due to its simple one‐component formulation. Preclinical studies have demonstrated that MonoSeal not only provides superior hemostatic efficacy and biocompatibility but also effectively seals both large (12 Fr) and clinically common (6 Fr) vascular punctures without leaving residual material or requiring external activation. These advantages address the critical limitations of existing vascular closure devices. MonoSeal shows promise as a reliable hemostatic solution for life‐threatening bleeding and post‐interventional hemorrhage, as well as serving as a closure device for large‐sized clinical punctures, offering a streamlined and practical alternative to current vascular sealing technologies.

## 5 Experimental Section

### Materials

The dextran was obtained from Shanghai Huamao Pharmaceutical Co. Sodium periodate (NaIO_4_) was purchased from Aladdin Biochemical Technology Co. Ethanol was purchased from Macklin Biochemical Technology Co. Fetal bovine serum (FBS), cell culture medium (L‐DMEM), and trypsin were purchased from Gibco.

### Preparation of MonoSeal

Weigh the polysaccharide accurately and prepare a homogeneous solution. Sodium periodate was added, and the reaction was carried out at room temperature protected from light. A drop of glycerol was added to stop the reaction, and excess ethanol was added to the reaction solution to precipitate the product, then carried out dehydration for 3–4 times, and the obtained product was dried in a vacuum drying oven at 40 °C to dry the oxidized polysaccharide. Dissolve the oxidized polysaccharide into a homogeneous solution at a certain concentration with pure water. Precisely measured volumes of a fixed solution and placed into a mold for freeze‐drying. Adhesive sponges were obtained as closures with body fluid‐triggered properties. Mainly the adhesive closure performance of MonoSeal was demonstrated using prepared ODex in the subsequent tests.

### Characterization of Functional Groups and Microstructure for Oxidized Polysaccharide‐Based Sponge

The main components and chemical bonding differences of the polysaccharide backbone were analyzed by ART‐FTIR, both pre‐and post‐oxidation, for the characterization of functional groups of the oxidized polysaccharide backbone. UV spectroscopy was used to analyze the aldehyde group content of products prepared with varying reaction ratios of polysaccharide to sodium periodate, aiming to identify the optimal conditions for oxidized polysaccharide preparation. SEM was employed to characterize the microstructure of MonoSeal, providing insights for optimizing its preparation process.

### Determination of Aldehyde Content and Oxidation Degree

The aldehyde content of the oxidized polysaccharide was determined by hydroxylamine hydrochloride titration. Briefly, hydroxylamine hydrochloride solution (0.25 mol L^−1^) was freshly prepared by dissolving 8.69 g of dried hydroxylamine hydrochloride in 75 mL of ultrapure water. Then, 3 mL of 0.05% (w/v) methyl orange solution was added as a pH indicator. The mixture was diluted to 500 mL with ultrapure water and mixed thoroughly. For the blank titration, 25 mL of the prepared hydroxylamine hydrochloride solution was transferred into a beaker and titrated with a 0.1 mol L^−1^ NaOH solution until the color transitioned from red to yellow. The consumed volume of NaOH was recorded as V_0_. For the sample titration, ≈0.5 g of the oxidized polysaccharide sample was accurately weighed and added to a beaker containing 25 mL of the hydroxylamine hydrochloride solution. The mixture was stirred at room temperature (≈25 °C) for 3 h to allow full reaction of aldehyde groups with hydroxylamine. After the reaction, the solution was titrated with 0.1 mol L^−1^ NaOH until the endpoint color transition from red to yellow was observed. The volume of NaOH consumed was recorded as V_1_. The aldehyde content (Av) was calculated as the millimolar amount of aldehydes per gram of oxidized polysaccharide.

(1) $$A_{v}=\frac{V_{1}-V_{0}}{W}\times M$$

The oxidation of aldehyde groups (Aw) is defined as the percentage of polysaccharide units that contain aldehyde groups. It could be calculated using the following formula:

(2) $$A_{w}=\frac{A_{v}\times M_{w}}{1000}\times 100\%\;=1.60\times \left(V_{1}-V_{0}\right)\%$$

Where:

V1: Volume of sodium hydroxide consumed by the sample solution (mL);

V0: Volume of sodium hydroxide consumed by the blank solution (mL);

M: Molar concentration of sodium hydroxide;

M_w_: Weight of monosaccharide units in the polysaccharide chain (the molecular weight of glucose units is 160 g mol^−1^);

W: Weight of oxidized polysaccharide (g).

### Verification of Chain penetration and Adhesion Mechanism via Fluorescent Labeling

It was hypothesized that MonoSeal forms strong adhesion on tissue surfaces primarily through a body‐fluid‐triggered chain penetration process, followed by covalent interfacial anchoring. To investigate the onset of chain penetration and chain interdiffusion, ODex was fluorescently labeled with Rhodamine‐PEG‐NH_2_. Specifically, 1.08 g of oxidized dextran was dissolved in 100 mL of 0.1 m acetic acid solution, and an equal volume of anhydrous methanol was added slowly while stirring. Rhodamine 110 chloride (44.02 mg) was dissolved in 44.02 mL of anhydrous methanol, stirred well, and then added dropwise to the oxidized dextran solution. The ratio of the amino group of Rhodamine B‐PEG‐Amine (RB‐PEG‐NH_2_) chloride to the aldehyde group of oxidized dextran was 1%. The reaction was carried out at room temperature and protected from light for 24 h. Precipitation of the oxidized dextran was carried out by adding the mixture drop by drop to five times ethanol. Repeat 3 to 5 times until the liquid was non‐fluorescent. The precipitate was dried and redissolved to 5% with deionized water. Lyophilization yielded the labeled sponge.

The fluorescently labeled MonoSeal was pressed onto fresh porcine skin and muscle, incubated at 37 °C for 1–2 h, and then frozen at ‐80 °C. Then the freezer performed a frozen section, and the obtained sections were analyzed under a fluorescence microscope to characterize the depth of ODex‐(RB‐PEG‐NH_2_) penetration into the tissues.

### Body Fluid‐Triggered Gelation Study

To investigate the body fluid‐triggered gelation performance of MonoSeal, equal weights of the lyophilized sponge were added to a fixed volume of different physiological fluids, including fresh blood, bile, pancreatic fluid, gastric fluid, and water. All fluids were prewarmed to 37 °C before testing to mimic physiological conditions. The onset of gelation, gelation rate, and appearance were observed at predetermined time intervals. Gelation was defined as the formation of a gel of material in body fluid without exhibiting dissolution or other phenomena. Each experiment was repeated 3 times to ensure reproducibility.

### In Vitro Adhesive Performance Evaluation

To assess the adhesive performance, the prepared MonoSeal was compressed onto the surface of various tissues (heart, liver, spleen, lung, kidney, stomach, small intestine, skin, rat tail, and rat dorsum. The adhesive strength was assessed by holding the materials with tweezers to lift the tissue.

### In Vitro Adhesive Performance Evaluation—Lap Shear Test

To perform the peel test, the porcine skin was cut into strips of 5.0 × 1.0 cm. Fresh blood was spread on the strip surface (1.0 × 1.5 cm) and then ODex, Dex with a thickness of 3.5 mm was pressed onto the surface of the two pork strips. The strips were placed in a universal mechanical tester once the gel was formed. Reverse tension was applied to the free end of the strips at a steady loading rate of 2 cm min^−1^. The shear strength of the MonoSeal was determined at the separation point. The test was repeated by applying commonly known commercial adhesives such as gelatin sponges and cyanoacrylates.

### In Vitro Adhesive Performance Evaluation—Tack Test

Protein casing with 2 × 2 cm sizes was adhered to the slide using cyanoacrylate adhesives. ODex and Dex sponges of 3.5 mm thickness were placed between slides covered with fresh blood. The samples were tested for adhesive strength by a universal mechanical tester at a steady loading rate of 2 cm min^−1^. The test was repeated by applying commonly known commercial adhesives such as gelatin sponges and cyanoacrylates.

### In Vitro Adhesive Performance Evaluation—Burst Pressure Test

Fresh pig sausage coating was cut into 4 × 4 cm sizes and fixed to a test device connected to a manometer. A hole with a diameter of 2 mm was formed in the center of the sausage coating. Fresh blood was spread on the coating's surface. MonoSeal and commercial adhesive (gelatin sponge) with a thickness of 5 mm were then applied to cover the defect. After gel formation, constant pressure was applied to the defect site by the microflow control system, and manometer readings were recorded. The maximum reading on the manometer before pressure loss was recorded as the burst pressure of the closure.

### Hemolysis Rate Test

To test the hemolytic activity of MonoSeal, fresh rabbit blood was centrifuged, and the erythrocytes were obtained after three washes with deionized water. The purified erythrocytes were diluted to a concentration of 10% (v/v). MonoSeal was diluted to 25% (w/v) concentration with deionized water. The ODex solution and erythrocyte suspension were mixed 1:1 and later incubated at 37 °C for 1 h. The samples were centrifuged, and the absorbance of the clear supernatant was measured at 540 nm using a microplate reader. Deionized water and saline were used as a positive control and a negative control, respectively. The test was repeated 3 times for each sample. The percentage hemolysis of ODex was calculated using the formula:

(3) $$Hemolysisratio\left(\%\right)=\frac{A_{h}-A_{p}}{A_{t}-A_{p}}\times 100\%$$

Here, *A*_h_、*A*_p,_ and *A*_t_ are the absorbance values of the supernatant fraction of the sample, negative control, and positive control, respectively.

### Toxicology and Cell Proliferation Assays

The cytotoxicity of MonoSeal was evaluated using the CCK‐8 assay to detect the proliferation of L929 cells. The materials were first soaked in anti‐coagulated porcine blood or 10% BSA at a ratio of 1 g:7 ml and allowed to gel completely. Afterward, the gelled material was cut into pieces and soaked in a serum‐containing medium at a ratio of 0.2 g ml^−1^ for 24 h to prepare the extract. This extract was then used to culture L929 cells for 24 h, and the proliferation of the cells was subsequently assessed.

### Rat Subcutaneous Implantation

Un‐cross‐linked MonoSeal in vivo was evaluated for local response and biodegradability by subcutaneous implantation in rats. Rats were anesthetized and shaved, and an incision was made on the back with scissors, and the subcutaneous tissue was separated to form a pocket. The prepared MonoSeal was put into the pocket directly. After suturing, the incision was disinfected, and the animal was put back on the thermostatic electric blanket to keep warm until the anesthetic effect wore off, and then put back into the cage. The degradation of the MonoSeal was observed. Once the time was reached, an excess of anesthesia was injected intraperitoneally, and the rats were euthanized. Samples were collected and the degradation of the material and the degree of inflammatory reaction around the material were observed.

### Rabbit and Porcine Liver Hemostasis

The hemostatic performance of MonoSeal with different preparation conditions was verified by using it in a rabbit liver hemorrhage model. A 1–1.5 cm long, 0.5 mm deep wound was prepared on the surface of the rabbit liver with a surgical knife. After bleeding, the prepared MonoSeal was pressed onto the wound. Thirty seconds later, whether there was blood oozing out was observed.

The hemostatic performance of the optimal MonoSeal was verified by the extreme bleeding model in the porcine liver. A 2 × 2 cm^2^ wound was created on the surface of the porcine liver using a surgical knife. After bleeding, the prepared MonoSeal (3 × 3 cm^2^) was pressed on the wound. Thirty seconds later, whether there was blood oozing out was observed.

### In Situ Degradation in Rabbit Liver

The actual degradation of MonoSeal after cross‐linking to form a hydrogel in body fluids was assessed by performing in situ degradation tests in the liver of rabbits. First, a wound 1–1.5 cm long and 0.5 mm deep was formed on the surface of the liver with a surgical scalpel. Prepared MonoSeal (1.5 × 1.5 cm^2^) was pressed over the wound after bleeding. After 30 s, it was observed whether there was any blood seepage or not. If not, the wound was closed, sutured and rearing continued. The rabbits were euthanized at each time point, and samples were collected. The degradation of the material and the adhesion of the surrounding tissues were observed, and histological analyses were performed.

### Lethal Hemorrhage Model of Porcine Femoral Artery

A large‐sized open puncture was modeled on the femoral artery of porcine. Briefly, after exposing the femoral artery, a 12 Fr vascular puncture sheath was used for the puncture intervention. Upon evacuation of the puncture sheath, a large amount of blood spurted out of the puncture opening. Pressure was applied proximally to expose the puncture opening to view. The prepared MonoSeal was pressed over the puncture wound, pressed for a certain period, and observed for bleeding. If there was none, the wound was closed, sterilized and rearing continued. Doppler ultrasound was performed on the femoral artery at 1, 7, and 14 days postoperatively to observe the patency of blood flow. After 14 days, the porcinis were euthanized, samples were collected, the repair of blood vessels was observed and histological analysis was performed.

### Post‐intervention Hemostasis Model of Porcine Carotid Artery

A puncture intervention was modeled on the carotid artery in porcine, and a vascular occluder designed was applied with a MonoSeal to close and arrest post‐puncture vessels. Briefly, the catheter sheath was retained after puncture intervention on the carotid artery with a vessel sheath set (6 Fr) through Doppler ultrasound‐guided puncture. A puncture intervention was modeled on the carotid artery in porcine, and a vascular occluder designed was applied with a MonoSeal to close and arrest post‐puncture vessels. Briefly, the catheter sheath was retained after puncture intervention on the carotid artery with a vessel sheath set (6 Fr) through Doppler ultrasound‐guided puncture. The matching occluder with MonoSeal was then inserted into the catheter sheath. All devices were withdrawn once the material was pushed to the blocking site by pressing the plug. Press the occlusion site and observe bleeding. If there was no bleeding, the wound was sterilized and feeding continued. Doppler ultrasound was performed on the carotid artery at 1, 7, and 14 days postoperatively to see whether the blood flow was smooth. CT of the carotid artery was performed 1 day after surgery to observe hematoma formation. After 14 days, the porcinis were euthanized, samples were collected, the vascular repair was observed and histological analysis was performed.

### Histological Analysis

The target areas at each time point were collected and fixed in 4% paraformaldehyde (PFA) before paraffin embedding and tissue sectioning. Histological analysis was performed by H&E staining to determine morphological changes in wound regeneration and quality of repair.

### Statistical Analysis

All data were presented as mean ± standard deviation (SD). Statistical comparisons between independent groups were performed using an independent samples t‐test to compare means. One‐way analysis of variance (ANOVA) was performed to assess statistical significance. Post‐hoc tests were applied where applicable to further analyze pairwise comparisons. The significance level was set at a p‐value of less than 0.05, which was considered statistically significant. All statistical analyses were performed using Origin software. Assumptions for the statistical tests, including normality and homogeneity of variances, were checked, and the tests used were deemed appropriate based on these assumptions.

### Ethical Approval

Thanks to the animals for their sacrifices. All the animal experiments involved in this work were approved by the Animal Ethics Committee of Zhejiang University (approval no. ZJU20240422).

## Conflict of Interest

The authors declare no conflict of interest.

## Author Contributions

R.W., W.Z., and Y.H. contributed equally to this work and are also co‐first authors. Thanks to the authors for their contributions. R.W., W.Z., Y.H., H.O., and Y.H. designed the study and wrote the manuscript. R.W., W.Z., Y.H., J.Y., and Y.Z. performed cell, histological, and animal experiments. R.W. and Y.H. designed and fabricated MonoSeal and performed material characterization. R.W., W.Z., Y.H., Y.G., S.X., Z.C., and Y.H. contributed to animal surgery and data collation. R.W., R.Z., and Y.H. have drawn. R.Z., T.Y., J.X., and H.S. contributed to data collation and analysis. Z.C., X.Z., and X.W. contributed to the data validation and review of the manuscript. H.O. and Y.H. supervised the study and contributed to the study design and review of the manuscript. All authors have read and agreed to the published version of the manuscript.

## Supporting information



Supporting Information

Supplemental Movie 1

Supplemental Movie 2

Supplemental Movie 3

Supplemental Movie 4

Supplemental Movie 5

Supplemental Movie 6

Supplemental Movie 7

Supplemental Movie 8

Supplemental Movie 9

## Data Availability

The data that support the findings of this study are available from the corresponding author upon reasonable request.
