# Supplementary material for: A Single‐Component Adhesive Sponge Based on Blood‐Triggered and Autopenetrative Adhesion for Robust Vascular Closure
Source: Adv Sci (Weinh). 2025 Aug 12;12(41):e10377. doi: 10.1002/advs.202510377 (PMC12591214; doi:10.1002/advs.202510377)
Supplement: Supplementary file 1 — Supporting Information [file ADVS-12-e10377-s010.docx]

**A Single-component Adhesive Sponge Based on Blood-Triggered and Autopenetrative Adhesion for Robust Vascular Closure**

**Rong Wang^1,2#^, Weishi Zheng^1,2#^, Yuxuan Huang^1,2#^, Rui Zhang^1,2^, Yi Zhang^1,2^, Jingying Yan^1^, Yuqing Gu^1,2^, Jingyun Xi^3,4^, Tun Yuan^3,4^, Hua Su^5^, Xianzhu Zhang^1,2^, Xiaozhao Wang^1,2^, Shaohui Xiong^6^, Zhenfeng Cheng^6^*****, Hongwei Ouyang^1,2,7^***, **Yi Hong^1,2,7^***

^1^ Department of Sports Medicine of the Second Affiliated Hospital, and Liangzhu Laboratory, Zhejiang University School of Medicine, Hangzhou, China.

^2^ Dr. Li Dak Sum & Yip Yio Chin Center for Stem Cells and Regenerative Medicine, Zhejiang University School of Medicine, Hangzhou, China.

^3^ National Engineering Research Center for Biomaterials, Sichuan University, Chengdu, Sichuan, China

^4^ Sichuan Testing Center for Biomaterials and Medical Devices Co., Ltd. Chengdu, Sichuan 610064, China

^5^ Department of Pulmonary and Critical Care Medicine, Regional Medical Center for National Institute of Respiratory Diseases, School of Medicine, Sir Run Run Shaw Hospital, Zhejiang University, No. 3 Qingchun Road East, Hangzhou, China.

^6^ Huzhou Central Hospital, Affiliated Central Hospital of Huzhou University, Huzhou, China.

^7^ China Orthopedic Regenerative Medicine Group (CORMed), Hangzhou, China.

#Co-ﬁrst author

*Corresponding authors

E-mail address: yihong@zju.edu.cn, [hwoy@zju.edu.cn,](mailto:hwoy@zju.edu.cn,) czf918@qq.com

## Supplementary Figures 1-9

**
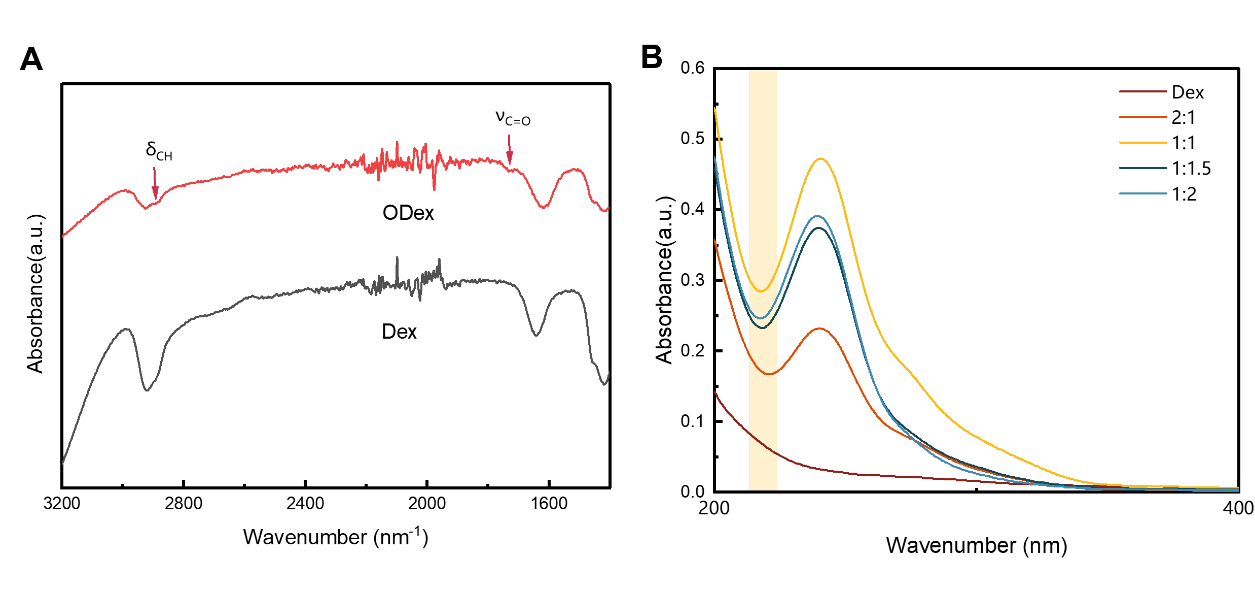
**

**Figure S1. Synthesization and characterization of ODex main chain.** A). ART-FTIR spectra of Dex and ODex. Distinct vibrational peaks were observed in the ATR-FTIR spectra of the oxidized skeleton at 2880-2650 cm^-1^ and 1740-1715 cm^-1^, compared to the absence of these peaks in the unoxidized skeleton, confirming successful periodate oxidation and aldehyde group introduction; B). UV spectra of Dex and ODex were prepared with different reaction ratios (The reaction ratios of Dex with sodium periodate were 2:1, 1:1, 1:1.5, and 1:2, respectively). All ODex samples exhibited a distinct aldehyde-associated absorption peak at 238 nm. Notably, the reaction ratio of 1:1 yielded the strongest absorbance at this wavelength, indicating the optimal incorporation of aldehyde functionalities under this condition.


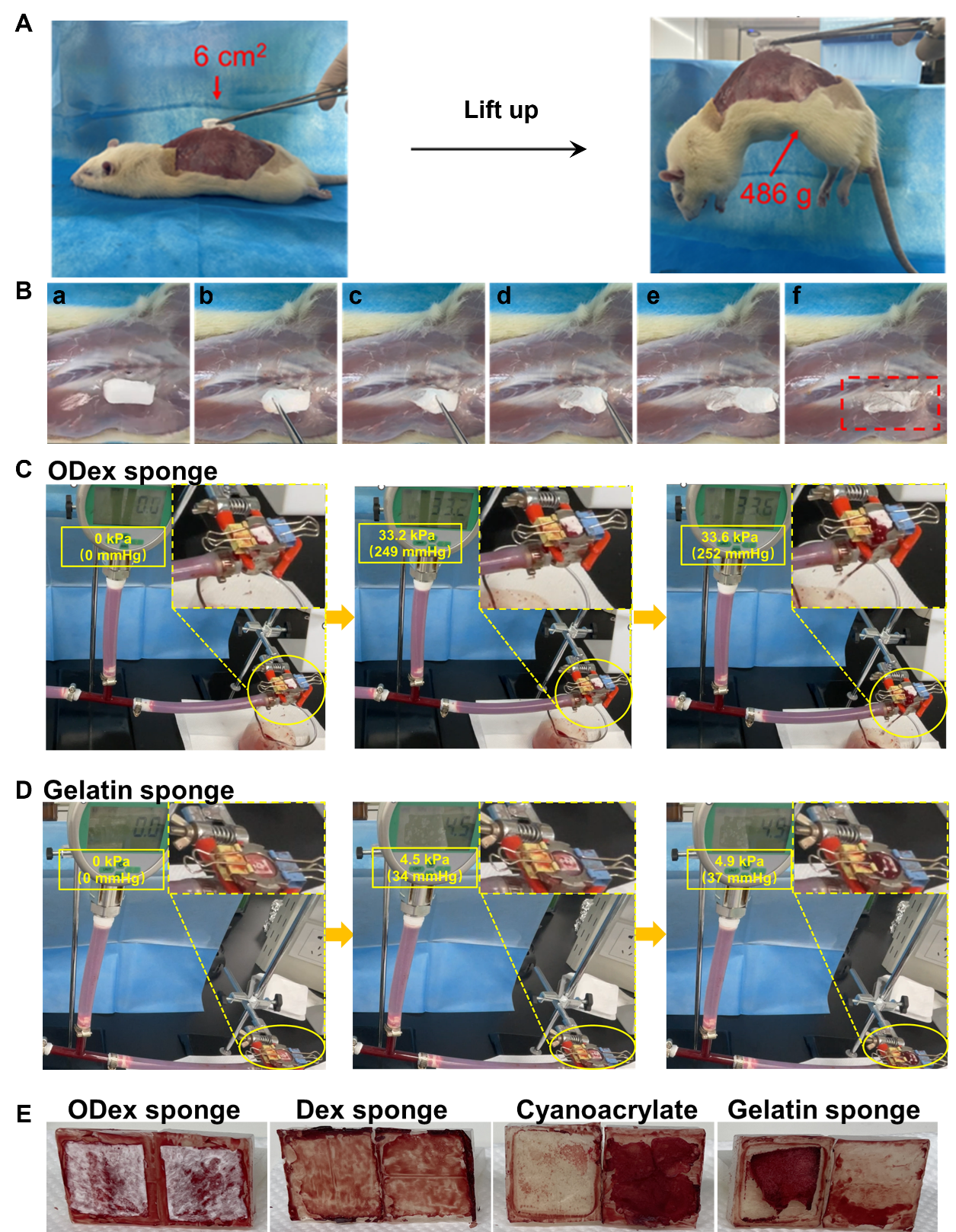


**Figure S2. Adhesion behavior and interfacial failure analysis of MonoSeal on wet tissues. A,B) MonoSeal exhibited strong tissue adhesion upon body-fluid contact.** A single sponge firmly adhered to the muscle surface of a 486 g rat, enabling complete body suspension (A). After mechanical peeling with tweezers (B), a residual adhesive layer remained on the tissue surface, visually indicating strong interfacial anchoring. **C,D) Burst pressure evaluation on simulated enteric defects (2 mm in diameter) under fresh blood exposure.** MonoSeal formed a tight seal, withstanding pressures up to 252 mmHg (C), exceeding both clinical systolic pressure thresholds (120-160 mmHg) and gelatin sponge performance (32 mmHg) (D). The sponge remained intact after test completion. **E) Representative failure modes captured post-tack test.** Dex sponge dissolved rapidly upon blood contact, resulting in negligible adhesion due to weak non-covalent interactions. Gelatin sponge and cyanoacrylate adhesives failed via interfacial detachment. In contrast, MonoSeal showed cohesive failure within the bulk matrix, with fracture occurring inside the sponge rather than at the interface. This confirms the formation of a densely crosslinked interfacial network via body-fluid-triggered chain penetration, establishing stable tissue anchorage even under stress.


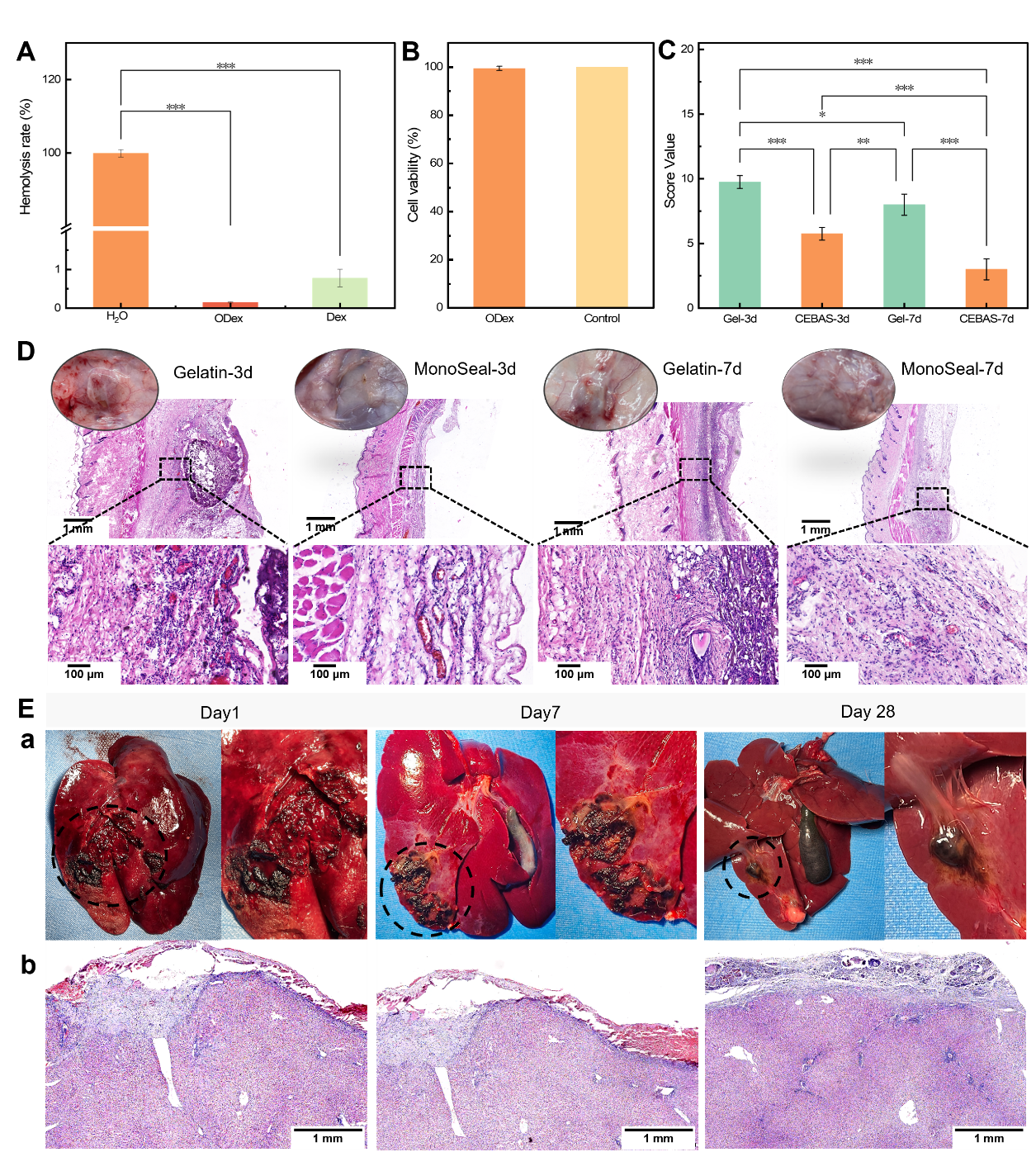


**Figure S3. Biocompatibility evaluation of MonoSeal *in vitro* and *in vivo*. A) Hemolysis assay.** MonoSeal exhibited excellent blood compatibility with no visible hemoglobin release; erythrocyte suspensions remained clear and transparent after incubation with MonoSeal-treated supernatant, indicating negligible hemolytic activity. **B) Cytocompatibility analysis.** L929 fibroblasts cultured with MonoSeal extract for 24 h showed no significant difference in cell viability compared to the untreated control, confirming minimal cytotoxicity. **C-E) *In vivo* degradation and biosafety of uncrosslinked MonoSeal.** (D) Non-gelled MonoSeal sponges and gelatin sponges were implanted subcutaneously in rats to assess foreign body response. (C) Semi-quantitative scoring of inflammatory cell infiltration on days 3 and 7 revealed mild and resolving inflammation, with no significant difference between groups, indicating good histocompatibility. **(E) *In vivo* degradation and biosafety of crosslinked MonoSeal.** Following liver surface application in rabbits, MonoSeal formed an in situ hydrogel barrier that gradually degraded over time. (a) Representative macroscopic images showed progressive material resorption and tissue integration. (b) Histological analysis of surrounding liver tissue demonstrated no significant necrosis or adverse inflammatory response during the degradation period (Data are presented as mean ± SD, ** p<0.01, *** p<0.001).


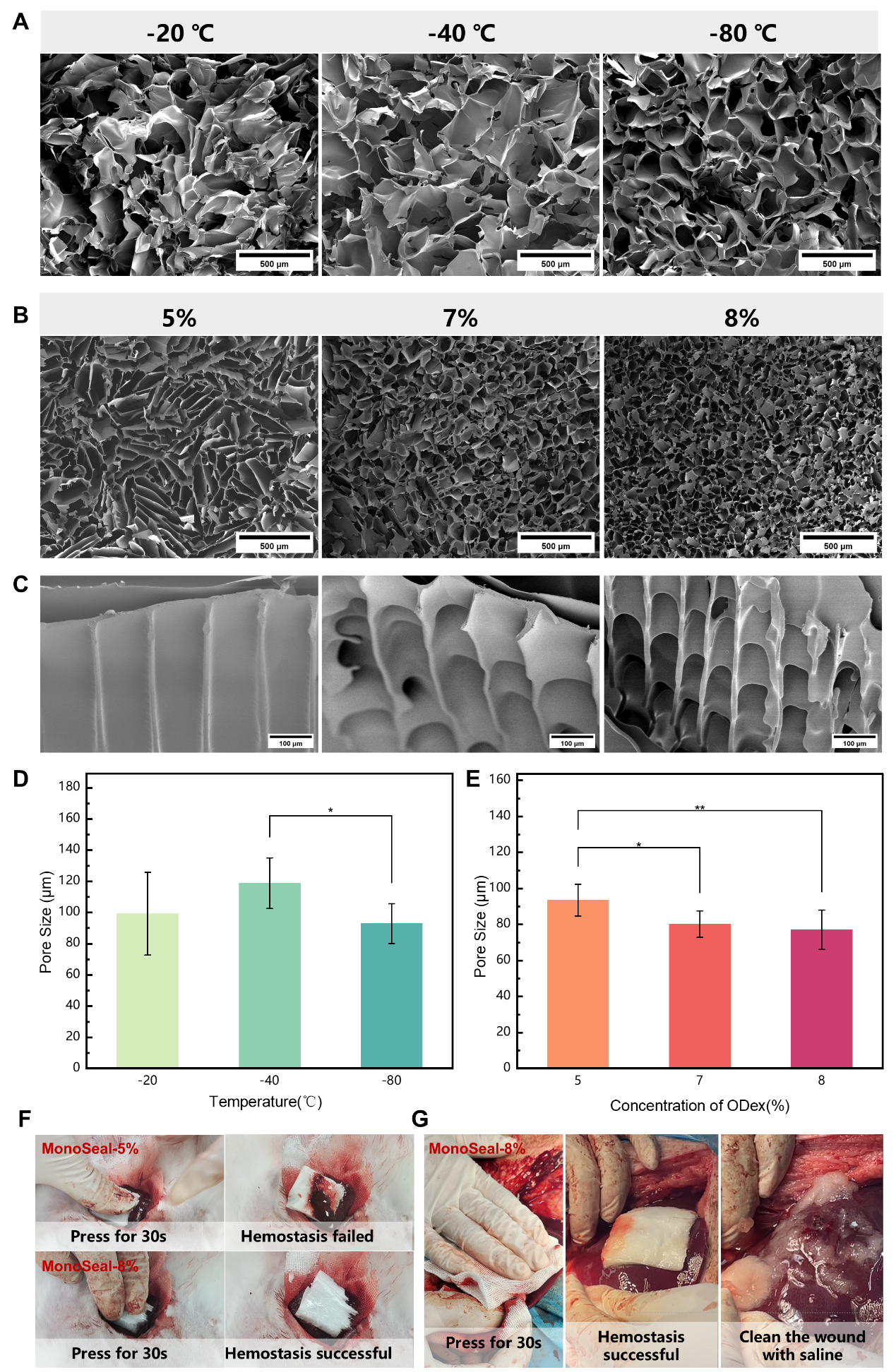


**Figure S4. Optimization of pore structure improves MonoSeal performance under high-pressure bleeding.** **A) Scanning electron microscopy (SEM) images of MonoSeal prepared at -20°C, -40°C, and -80°C under 4% ODex concentration.** Among them, the -80°C group exhibited the most uniform and compact porous architecture and was selected as the optimal pre-freezing condition. **B,C) SEM characterization of MonoSeal sponges lyophilized at -80°C under varying ODex concentrations (5%, 7%, 8%)**. Increasing polymer concentration reduced pore size and enhanced pore density (B) while promoting the emergence of transverse wave-like microstructures that resisted vertical fluid infiltration (C). **D,E) Quantitative analysis of pore size distribution in MonoSeal sponges fabricated under the above conditions** (* p<0.05, ** p<0.01)**.** 7% and 8% ODex groups showed comparable structural features and were considered optimal for further validation. **F,G) In vivo evaluation of MonoSeal sealing performance in rabbit (F) and porcine (G) liver hemorrhage models.** MonoSeal prepared at -80°C/5% failed to achieve hemostasis due to directional pore alignment and insufficient structural resistance. In contrast, MonoSeal optimized at -80°C/8% demonstrated rapid and effective sealing in both models, attributed to its compact pore network and transverse barrier morphology that provided sufficient time for ODex chain dissolution, diffusion, and gelation. These results highlight the importance of freeze-drying control in tailoring sponge microarchitecture to enhance functional sealing performance.


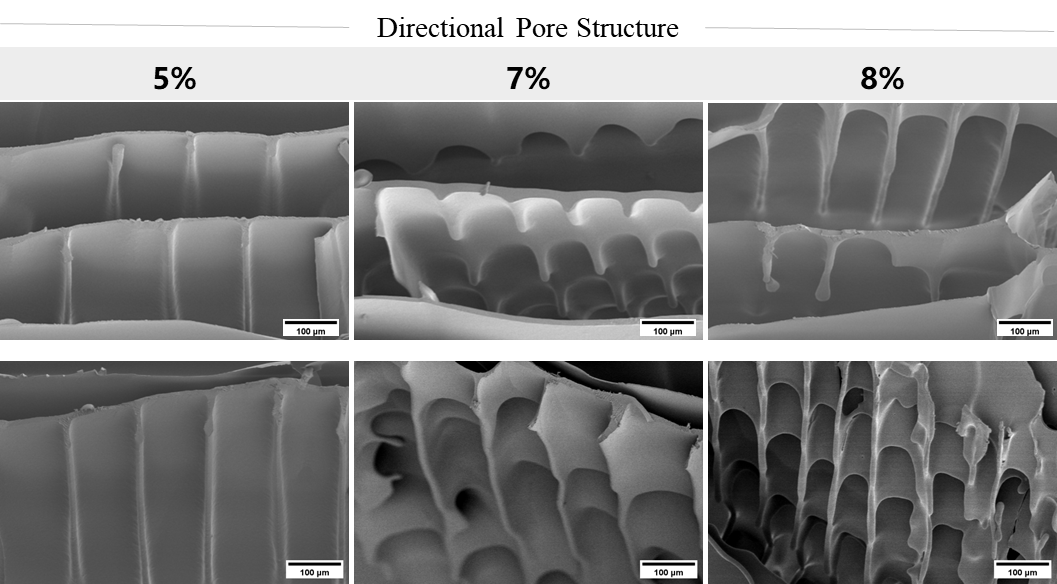


**Figure S5. Enhanced SEM imaging of MonoSeal sponge at varying ODex concentrations and different angles.** SEM images showing MonoSeal sponges lyophilized at -80°C under different ODex concentrations (5%, 7%, and 8%). The images provide additional angles to further illustrate the anisotropic pore alignment and the formation of transverse wave-like microstructures in the sponges. These transverse features are particularly prominent in higher polymer concentrations (7% and 8%), which enhance the material's resistance to vertical blood infiltration. These further confirmation of how the pore architecture contributes to the efficient gelation and sealing process, ensuring better mechanical sealing performance during high-pressure bleeding conditions.**
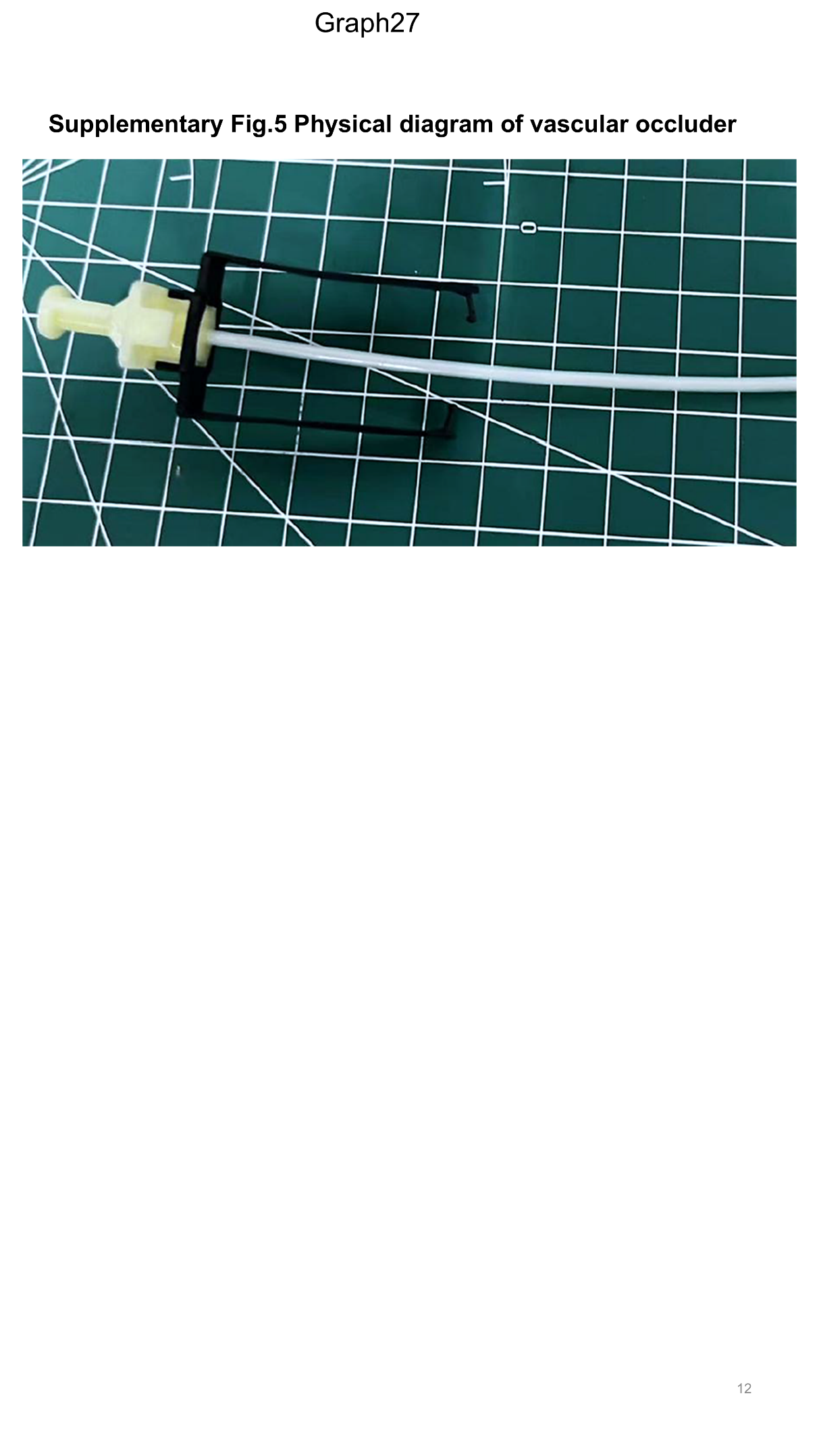
**

**Figure S6.** Physical diagram of the vascular occluder.


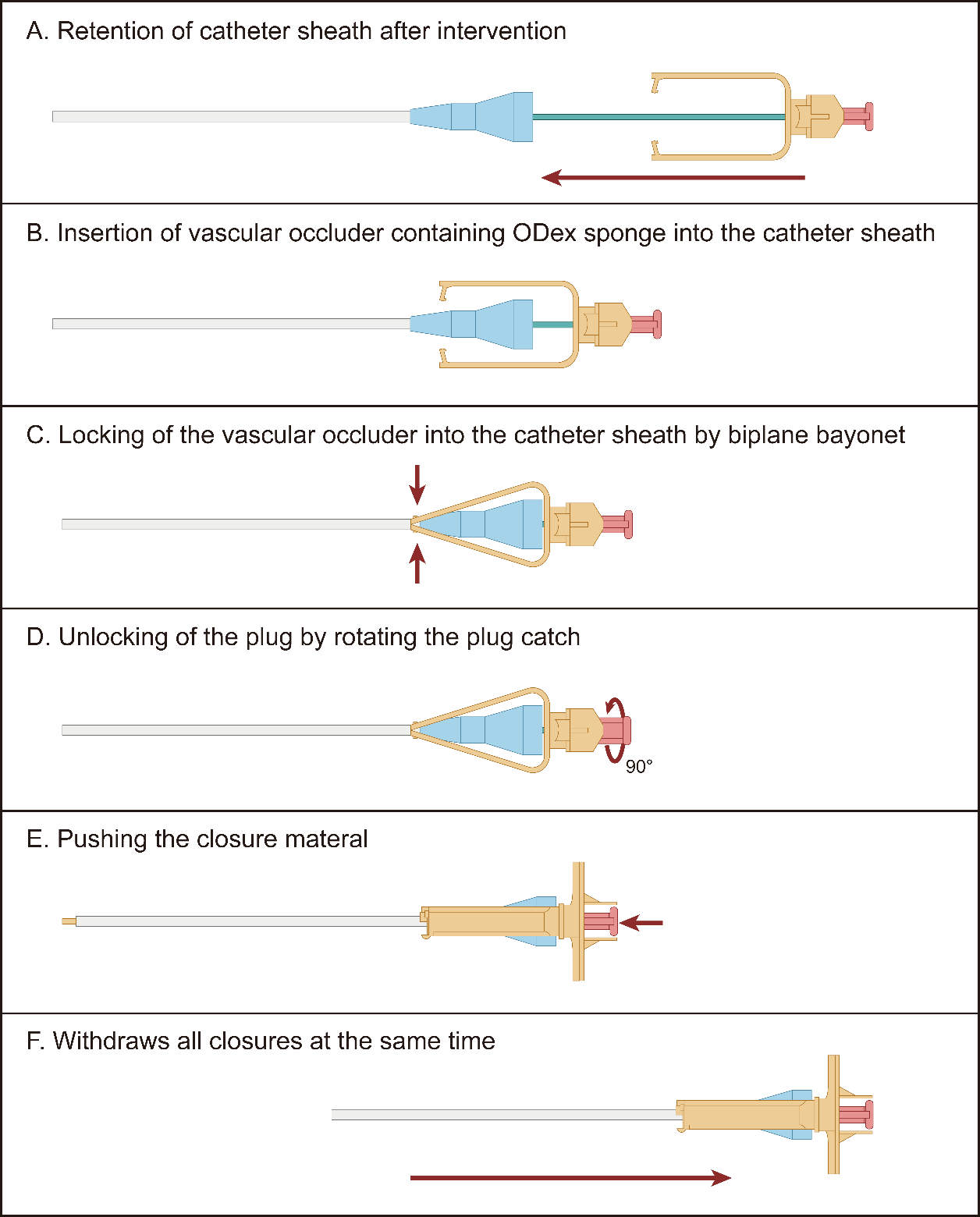


**Figure S7. Vascular occluder and the method of its use.** A) Retention of catheter sheath after intervention, showing the stable positioning of the catheter sheath within the vessel. B) Insertion of the vascular occluder containing the ODex sponge into the catheter sheath, ensuring accurate placement for occlusion. C) Locking mechanism of the vascular occluder into the catheter sheath using the biplane bayonet, providing secure attachment for effective sealing. D) Unlocking the plug by rotating the plug catch, allows easy removal of the occluder after the sealing process is complete. E) Pushing the closure material into the catheter sheath facilitates the occlusion process with MonoSeal for hemostasis. F) Withdrawal of all closure components simultaneously, demonstrating the efficiency and simplicity of the vascular occlusion and closure procedure.


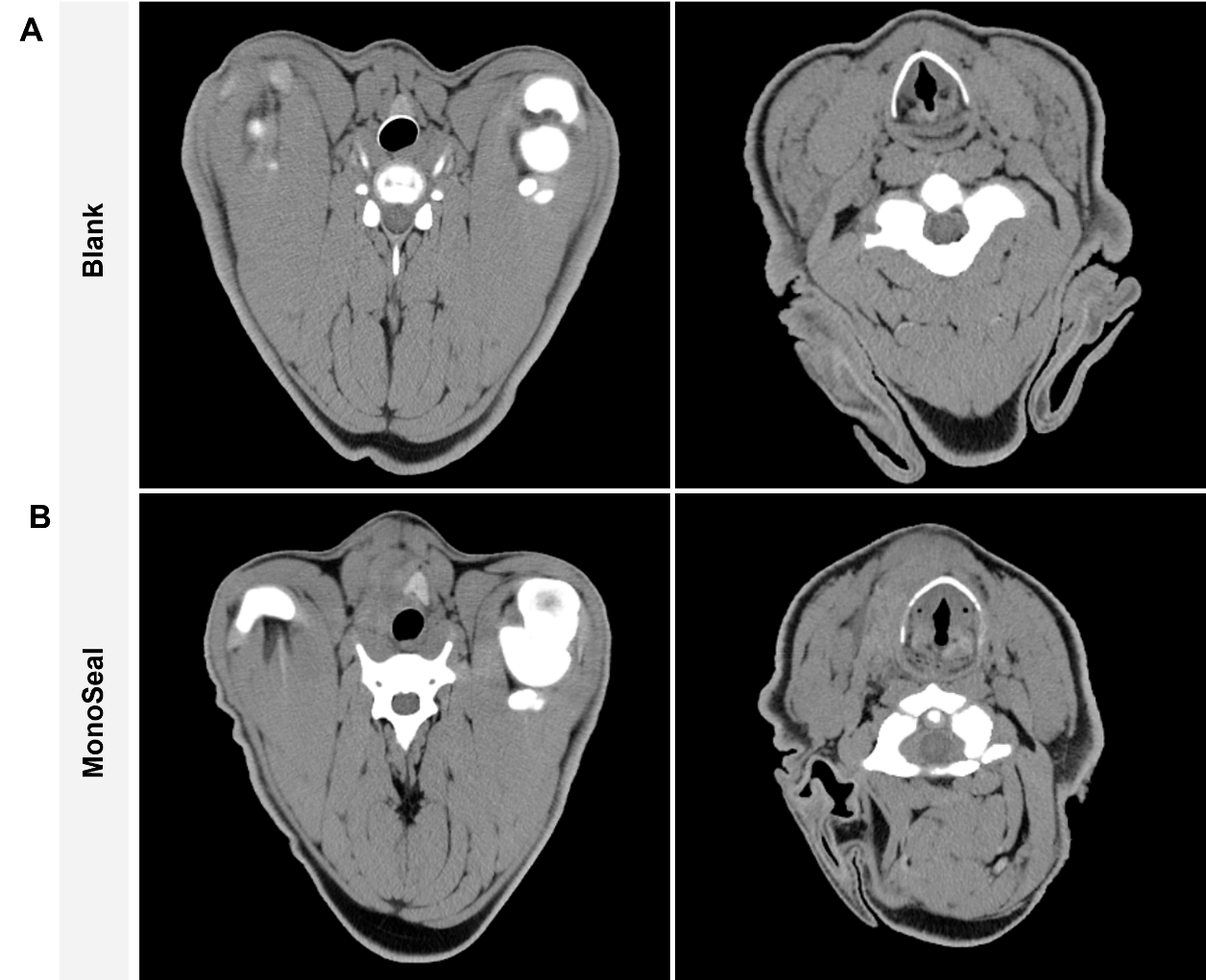


**Figure S8. CT test for postoperative hematoma formation in porcine carotid arteries.** There was no significant hematoma formation in the porcine carotid artery following ODex sponge treatment compared with the blank group, indicating effective sealing without adverse bleeding outcomes.


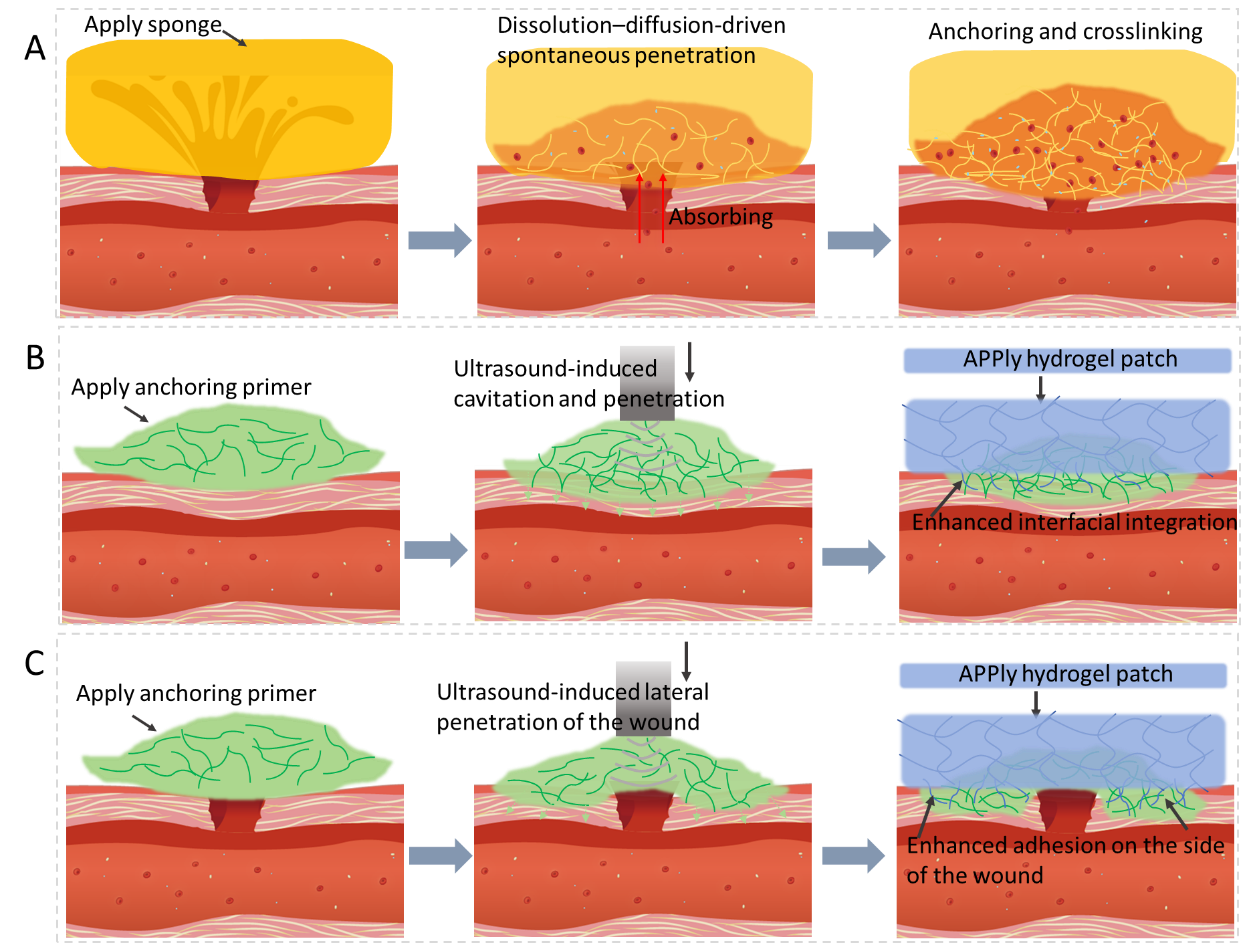


**Figure S9. Comparative analysis of the Autopenetrative Adhesion (APA) mechanism and existing chain penetration-enhanced adhesion strategies** (e.g., ultrasound-triggered methods). A) Schematic illustration of the APA mechanism, which operates solely through body-fluid-triggered polymer chain dissolution and spontaneous tissue penetration without the need for external stimuli. The polymer chains dissolve and automatically penetrate tissue structures, enhancing tissue interaction and forming covalent cross-links at both surface and subsurface levels to ensure robust adhesion under physiological conditions. B) Comparison with traditional methods, such as ultrasound -triggered strategies, which require external energy inputs to increase tissue permeability and drive polymer diffusion into tissues. C) The traditional method, such as ultrasound, cannot cause the polymer chains to penetrate at the wound tissue. This highlights the key distinctions: (1) Energy-free activation in APA versus external force or energy dependency; (2) Spontaneous diffusion of polymer chains under normal physiological conditions in APA; (3) Covalent interlocking in APA at deeper tissue levels compared to superficial interaction in traditional methods.

## Supplementary Movies 1-9

**Supplementary** **Movie 1.**

Peeling the MonoSeal sponge firmly adhered to the surface of the rat.

**Supplementary Movie 2.**

Burst pressure test of MonoSeal.

**Supplementary Movie 3.**

Burst pressure test of gelatin sponge.

**Supplementary Movie 4.**

Optimized MonoSeal for hemostasis of rabbit liver bleeding.

**Supplementary Movie 5.**

Optimized MonoSeal for hemostasis of porcine liver hemorrhage.

**Supplementary Movie 6.**

Extreme hemorrhage was simulated in porcine femoral arteries by employing a large-size (12 Fr) vascular sheath set, and MonoSeal was performed for hemostasis.

**Supplementary Movie 7.**

Clinically real vascular interventional puncture procedures were simulated in the porcine carotid artery, using a 6 Fr vascular sheath set, and hemostasis was performed by use of MonoSeal and accompanying vascular occluder.

**Supplementary Movie 8.**

Patency of the porcine femoral artery was tested at 1 day postoperatively, using Doppler ultrasound.

**Supplementary Movie 9.**

Patency of porcine carotid arteries at 1-day postoperatively, using Doppler ultrasound.
